# *Yap1* modulates cardiomyocyte hypertrophy via impaired mitochondrial biogenesis in response to chronic mechanical stress overload

**DOI:** 10.7150/thno.74563

**Published:** 2022-10-03

**Authors:** Peng Yue, Yue Zhang, Lei Liu, Kaiyu Zhou, Shutao Xia, Mou Peng, Hualin Yan, Xiaoqiang Tang, Zhan Chen, Donghui Zhang, Junling Guo, William T. Pu, Yuxuan Guo, Yimin Hua, Yifei Li

**Affiliations:** 1 Key Laboratory of Birth Defects and Related Diseases of Women and Children of MOE, Department of Pediatrics, West China Second University Hospital, Sichuan University, Chengdu, Sichuan 610041, China.; 2 State Key Laboratory of Biocatalysis and Enzyme Engineering, School of Life Science, Hubei University, Wuhan, Hubei 430062, China.; 3 Department of Medical Ultrasound, West China Hospital, Sichuan University, Chengdu, Sichuan 610041, China.; 4 Peking University Health Science Center, School of Basic Medical Sciences, The Institute of Cardiovascular Sciences, Key Laboratory of Molecular Cardiovascular Science of Ministry of Education, Beijing Key Laboratory of Cardiovascular Receptors Research, Beijing, 100191, China.; 5 BMI Center for Biomass Materials and Nanointerfaces, College of Biomass Science and Engineering, Sichuan University, Chengdu, Sichuan 610065, China.; 6 Department of Cardiology, Boston Children's Hospital, Boston, MA 02115 USA.; 7 Harvard Stem Cell Institute, Harvard University, Cambridge, MA 02138 USA.

**Keywords:** YAP signaling, pathological hypertrophy, mitochondria, Verteporfin

## Abstract

**Rationale:** Chronic pressure overload is a major trigger of cardiac pathological hypertrophy that eventually leads to heart disease and heart failure. Understanding the mechanisms governing hypertrophy is the key to develop therapeutic strategies for heart diseases.

**Methods:** We built chronic pressure overload mice model by abdominal aortic constriction (AAC) to explore the features of Yes-associated protein 1 (YAP1). Then AAV-cTNT-Cre was applied to *Yap1^F/F^* mice to induce mosaic depletion of YAP1. *Myh6^CreERT2^; H11^CAG-LSL-YAP1^* mice were involved to establish YAP1 overexpression model by Tomaxifen injection. ATAC-seq and bioChIP-seq were used to explore the potential targets of YAP1, which were verified by a series of luciferase reporter assays. *Dnm1l* and *Mfn1* were re-expressed in AAC mice by AAV-cTNT-Dnm1l and AAV-cTNT-Mfn1. Finally, Verteprofin was used to inhibit YAP1 to rescue cardiac hypertrophy.

**Results:** We found that pathological hypertrophy was accompanied with the activation of YAP1. Cardiomyocyte-specific deletion of *Yap1* attenuated AAC-induced hypertrophy. Overexpression of YAP1 was sufficient to phenocopy AAC-induced hypertrophy. YAP1 activation resulted in the perturbation of mitochondria ultrastructure and function, which was associated with the repression of mitochondria dynamics regulators *Dnm1l* and *Mfn1*. Mitochondrial-related genes *Dnm1l* and *Mfn1*, are significantly targeted by TEAD1/YAP complex. Overexpression of *Dnm1l* and *Mfn1* synergistically rescued YAP1-induced mitochondrial damages and cardiac hypertrophy. Pharmacological repression of YAP1 by verteporfin attenuated mitochondrial damages and pathological hypertrophy in AAC-treated mice. Interestingly, YAP1-induced mitochondria damages also led to increased reactive oxidative species, DNA damages, and the suppression of cardiomyocyte proliferation.

**Conclusion:** Together, these data uncovered YAP signaling as a therapeutic target for pressure overload-induced heart diseases and cautioned the efforts to induce cardiomyocyte regeneration by activating YAP.

## Introduction

Hypertensive heart disease is a major health problem caused by the prolonged elevation of blood pressure. High blood pressure results in cardiac pressure overload and triggers pathological hypertrophy 1. While short-term and moderate hypertrophy is often believed adaptive and compensatory, prolonged hypertrophy eventually becomes maladaptive and leads to decompensation and heart failure 2. The mechanisms by which pressure overload stimulates cardiac hypertrophy and causes myocardial damages are not completely understood. Over the past decade, heart failure with preserved ejection fraction (HFpEF) has become a major topic in heart failure research. HFpEF is a kind of functional compensation and pathological myocardial hypertrophy is one of the dominant changes that occur following HFpEF. However, without termination or reversal of myocardial hypertrophy and the associated injuries, HFpEF can progress to heart failure with reduced ejection fraction (HFrEF). This sets a major obstacle for the development of better therapeutic strategies for HFpEF.

A characteristic feature of cardiac hypertrophy is the thickening of ventricular walls and the enlargement of cardiomyocytes. As a result of these morphological changes, cardiomyocyte mitochondria undergo transcriptional, ultrastructural, and functional remodeling 3. Mitochondrial ultrastructure and function are tightly controlled by their fusion-fission dynamics 4. Mitofusin-1 (MFN1) is a core mitochondria fusion regulator. *Mfn1* is downregulated during cardiac hypertrophy 5, 6. Furthermore, the cardiac ablation of Mfn1 together with mitofusion-2 (MFN2), a redundant fusion regulator, cause mitochondria dysfunction, cardiomyocyte enlargement, and lethal cardiomyopathy 7, 8. Dynamin-1-like protein (DRP1, encoded by *Dnm1l* gene) is a major mitochondrial fission factor, the activation of which is implicated in the aggravation of cardiac hypertrophy and heart failure 9. In addition, DRP1 reduction is also reported to cause mitochondrial damage and promote hypertrophy 10. Accumulated evidences indicate that the dynamic balance between mitochondrial fusion and fission determines cardiac phenotypes 8. Imbalanced mitochondrial dynamics causes more extensive cardiac damages. This evidence implies that it is critical to identify the upstream regulators of mitochondrial dynamics in order to protect the heart against hypertrophy and mitochondria-related damages.

Yes-associated protein 1 (YAP1) is a transcription cofactor that plays essential roles in the control of organ sizes. In cardiomyocyte, YAP1 has been extensively studied in cardiomyocytes as a mitogenic factor that can promote cell cycle progression and myocardial regeneration 11, 12. Despite the promising therapeutic potential of YAP1-based therapy, the negative impact of YAP1 activation on the heart is becoming apparent. For example, animal studies have shown that YAP1 overexpression and hyperactivation in the heart can trigger ventricular wall thickening, cardiac pathogenesis, and even death 13, 14. Upon hypertensive stress induction by transverse aortic constriction (TAC), endogenous YAP1 activation was also reported to drive cardiac hypertrophy, which rapidly progressed into heart failure 15. Together, these studies imply that, depending on the developmental and pathological conditions, YAP signaling plays seemingly divergent roles in cardiac repair and pathogenesis. Further investigation of the mechanisms by which YAP1 functions in the heart is necessary to reconcile these data and enable YAP1-dependent cardiac therapy.

Studies of pressure overload-induced pathological hypertrophy are limited by the available methodology. Although TAC surgery can be used to trigger a profound hypertrophic response in murine hearts, this process is too rapid to fully represent the more chronic and progressive responses observed in human subjects, which usually lead to HFrEF. In this study, we attempted to solve this problem by performing abdominal aortic constriction (AAC) in mice 16 to investigate the mechanisms that drive chronic pathological hypertrophy with HFpEF in mice. Strikingly, we found chronic YAP1 activation perturbed the synergy between mitochondrial fusion and fission and promoted pathological hypertrophy. YAP1-induced mitochondrial damages eventually inhibited the initial mitogenic functions of activated YAP1. These data indicated that a precise temporal control of YAP1 activation is key to achieving successful cardiac regenerative therapy.

## Methods and materials

### Animal procedures

All animal procedures were performed following protocols approved by the Institutional Animal Care and Use Committee of West China Second University Hospital, Sichuan University. *Yap1^F/F^* 17, *Myh6^CreERT2^* 18, AAV-cTNT-GFP and AAV-cTNT-Cre-GFP 19 were described previously. *Dnm1l* and *Mfn1* coding sequence was PCR-amplified from plasmid containing CDS and inserted into AAV-cTNT-3XHA-P2A-GFP and AAV-cTNT-3XHA-P2A-mScarlet through *NheI* and *SacI* sites to generate AAV-cTNT-Dnm1l-P2A-GFP and AAV-cTNT-MFN1-mScarlet. *Yap1^F/F^* mice were purchased from Jax Lab (Jax, 027929). *H11^CAG-LSL-YAP1^* mice were constructed by Nanjing Gembio Co., Ltd (T010798).

Associated adeno virus (AAV) was injected subcutaneously at P1 or P28 at 4.0×10^10^ vg/g (high dose) or 2.0×10^10^ vg/g (low dose). Tamoxifen was administered intraperitoneally at 0.2 mg/g per day in one daily injection for 5 consecutive days. Verteporfin (Sigma, SML0534) was injected intraperitoneally at 0.1 mg/g for 10 consecutive days. Due to ketamine is strictly controlled in China, and it is difficult to be obtain for experiments, even in clinic. So that we selected tribromoethanol as the anesthetic reagent. Compared to ketamine, tribromoethanol did not increase the risk mortality and respiration inhibition in our lab. Furthermore, it would alleviate abdominal adhesions or inflammatory responses during surgery 20. At the same time, it has been reported that tribromoethanol has been used in aortic surgery 21. So during surgery, mice were anesthetized with a formula with pharmaceutical-grade tribromoethanol (1 g), tert-amyl alcohol (0.625 ml) and normal saline (50 ml) by intraperitoneal injection as a dose of 15-20 ul/g. The mice were anesthetized with isoflurane during echocardiography, using a mixture of isoflurane and oxygen at a concentration of 1-3% (up to 5% during induction) for inhalation anesthesia.

The AAC surgery was performed on 1 month mice. A longitudinal incision around 1 cm of the skin of abdomen was made with scissors below the sternum. After the abdomen cavity was opened, abdominal intestine was taken out and wrapped with gauze soaked in warm saline. Isolated abdominal aorta, locating at the left of the inferior vena cava. Placed 0.26 mm ligation pole parallel to the abdominal aorta, and tied around the aorta and ligation pole using suture. Removed the ligation pole immediately to create a lumen with a fixed stenotic diameter. The abdominal cavity was closed, and the mice were given intramuscular injection of 10 to 20 thousand units of penicillin for three days to prevent infection.

Echocardiography was performed on a VisualSonics Vevo 3100LT. Animals were wake during this procedure and held in a standard handgrip. Echocardiography was performed blinded to all groups.

### NMVM culture

NMVMs were isolated using the Neomyt Kit (Cellutron, NC-6031) and plated on 1% Matrigel (Corning, 354234)-coated plates in cardiomyocyte culture media (low glucose DMEM (Gibco), 5% horse serum (American Type Culture Collection, 30-2040), 2% chicken embryo extract (VWR, 100356-958)). The following day (day 1), adenoviral vectors (AdVs) (AdV-TnT-Cre, AdV-TnT-LacZ, AdV-TnT-Dnm1l-GFP, AdV-TnT-Mfn1-GFP, Ad-TnT-GFP) were added at a multiplicity of infection (MOI) of 20, which transduced >90% cTNT+ cells. The medium was changed daily to maintain the cells. SiRNAs were purchased from Guangzhou Ribobio Co., Ltd, and transfected into NVMVs with RNAiMax reagents (Invitrogen). Verteporfin (Sigma, SML0534) was administered at 10 µM. NMVMs were harvested at 5th or 7th culture day.

### Human iPSC, EHT (Engineered heart tissues) fabrication and stretch culture

The WTC human induced pluripotent stem cell (WTC-iPSC), which was described in previous research 22, was maintained in mTeSR1 (STEMCELL Technologies, 85850) and passaged by versene solution (Gibco, 15040-066). The differentiation protocol of hiPSC-cardiomyocytes were chemically-defined 23. HiPSC were treated with 7.5 µM CHIR99021 (STEMCELL Technologies, 72054) in differentiation medium (RPMI-1640 medium (Gibco, 61870-036) with B27 (Basal Media, X064C4). After 48 hours, medium was changed into 1640/B27. At day 3, cells were cultured in differentiation medium containing 5 µM IWR (Tocris, 3532-50) for another 2 days, then change differentiation medium every two day until day 15.

HiPSC-cardiomyocytes were dissociated to single cell using 0.2% collagenase type I (Sigma, 0130) for 1 hours and 0.25% trypsin/EDTA (Gibco, 25200056) for 5 minutes. Mixed 1×10^6^ cardiac single cells with 120 μL Hydrogel solution (2.4 μL 50 U/mL thrombin (Sigma, T7201), 24 μL 10 mg/mL fibrinogen (Sigma, F3879), 12 μL Matrigel (Corning, 354277), 58 μL cardiac media, and transferred to bundle molds (14×12 mm^2^ polydimethylsiloxane (PDMS, Dow Corning, SYLGARD184) molds with Velcro frame (1.2 mm long)), then put it at 37 °C, wait for it to solidify and form hEHT. After an hour, add 1.5 ml the hEHT medium (RPMI 1640 medium with 1 mM sodium pyruvate (Gibco, 11360070), 0.45 μM 1-Thioglycerol (Sigma, M6145), 0.1 mM non-essential amino acids (Gibco #11140050), 2 mg/mL 6-Aminocaproic acid (Sigma, A2504), 0.4 mg/mL Ascorbic Acid (Sigma, A4544), 1% Penicillin-Streptomycin (Gibco, 15140122)) supplemented with 5% FBS (ABW, LT22906) and changed every two days.

After 3 days of hEHT culture, it was transferred to a 12-well plate covered with 0.5 ml PDMS. The original length of the hEHT was 8 mm. One end of the hEHT was fixed with Minutien Pins (Fine Science Tools, 26002-20), and the other end was fixed after stretching 1 mm.

### Histology

Hearts were harvested immediately after mice were euthanized by CO_2_, and the tissues were fixed by 4% paraformaldehyde overnight at 4 °C. Fixed cardiac tissues were cryoprotected by soaking in 30% sucrose for 2-4h at room temperature. The tissues were embedded in optimal cutting temperature (OCT) compound (SAKURA, Tissue-Tek). Six micrometers of cryo-sections were cut using a cryostat (Leica, CM3050). HE (G1120, Solarbio, China) and Masson staining (G1343-7, Solarbio, China) were performed per the manual. The ratio of interstitial fibrosis area to the total left ventricular area was calculated from 15 randomly selected microscopic slides in individual sections per heart using a camera attached to a Leica DM2000 microscope, with images further analyzed by ImageJ, excluding coronary vessels and perivascular regions.

### Immunofluorescence

Tissues were fixed in 4% paraformaldehyde overnight, cryoprotected by overnight incubation in 30% sucrose, and embedded in tissue freezing medium (VWR, 25608-930). 8 µm cryosections were obtained using a cryostat (Thermo Scientific, Micron HM 550). Sections and cells were incubated in blocking medium (PBS containing 5% donkey serum, 0.2% Triton X-100) at 4 °C overnight. For immunostaining, sections were incubated with primary antibodies at 4 °C overnight and secondary antibodies for 2 h. Primary antibodies used were as follow: aYAP1 (1:200, Abcam, ab205270), Ki67 (1:200, Abcam, ab16667), γ-H2AX (1:200, Abcam, ab81299), ACTN2 (1:200, Abcam, ab137346), cardiac troponin I (1:200, Abcam, ab56357), ATP5B (ATP synthase, H^+^ transporting mitochondrial F1 complex, beta subunit; 1:200, Abcam, ab14730), MFN1 (1:200, Abcam, ab104274), DRP1 (1:200, ProteinTech, 12957-1-AP), and 4HNE (1:200, Abcam, ab46545). After washing with blocking buffer, the slices were incubated with optimal secondary antibodies with/without WGA and/or DAPI at room temperature for 2h. After washing, samples were mounted with ProLong Diamond antifade mountant (Invitrogen, 36961) and imaged using an Olympus FV1000 confocal microscope. Laser scanning confocal microscope (Olympus, FV1000) were taken to determine confocal fluorescence images. Fluorescence intensity and cell size were measured by ImageJ.

### Mitochondrial measurements

Hearts were fixed with glutaraldehyde-formaldehyde (2% formaldehyde and 2.5% glutaraldehyde in 0.1 M Sodium Cacodylate buffer, pH 7.4) overnight. Sections were imaged using a H-7650 (Hitachi, Japan) electron microscope (Sichuan University EM core facility). Mitochondrial size and density were measured using ImageJ.

Mitochondrial function was measured using a Seahorses Biosciences XFe analyzer. Adult cardiomyocytes were measured immediately after cell isolation. NMVMs were isolated and seeded in CM culture media in 1% Matrigel-coated Seahorse BioSciences XFe assay wells, cultured for 24h before AdV or chemical treatments. Then, culture medium was changed daily for 5 days. Oxygen consumption rates (OCRs) were measured using the Cell Mito Stress Kit (Seahorses Biosciences, 103015). Followed the protocol of Seahorse Cell Mito Stress Kit, the final concentration of Oligomycin was 1.5 µM, the final concentration of FCCP was 2.5 µM, and the final concentration of rotenone/antimycin A was 0.5 µM. Data were normalized to cell number measured by DAPI staining on culture plates.

Mitochondrial morphology was described using MitoTracker (Invitrogen, M7514 and M22426). MiNA (mitochondrial network analysis, https://github.com/StuartLab) with Fiji software was used to analyze the morphology of mitochondria. The tool incorporates optional preprocessing steps to enhance the quality of images before converting the images to binary and producing a morphological skeleton for calculating nine parameters to quantitatively capture the morphology of the mitochondrial network. 24. CellROX (Invitrogen, C10444) were used to measure the reactive oxygen species (ROS) levels by fluorescence intensity via confocal scanning microscopy. *In vitro* cultured *H11^CAG-LSL-YAP1^* cardiomyocytes treated with Ad:Cre or Ad:LacZ were staining with CellROX Green (5 µM; Thermo Scientific, C10444) at 37 °C for 0.5 hour and then washed three times with PBS. Cells were lifted for flow cytometry using 0.25% trypsin, then pelleted and resuspended in 200 µl culture medium. As a positive control, cardiomyocytes were also treated with 50 µM H_2_O_2_ for 10 min.

### *In situ* myocardial imaging and cardiomyocytes isolation

100 µg/ml FM 4-64 (Invitrogen, 13320) membrane dye was perfused in the heart for 10 min at room temperature through a Langendorff apparatus. The whole heart was then positioned on an inverted Olympus FV1000 confocal microscope as described previously 19. CM isolation for immunofluorescence analyses was performed as previously described 19. In brief, collagenase (Worthington, LS004176) was perfused throughout the heart using a Langendorff apparatus at 37 °C for 10 min. Cardiomyocytes were next mechanically dissociated into a single cell suspension and briefly cultured on laminin-coated coverslips for 30 min.

### Flow cytometry

By two months after AAV injection in sham, AAC or *Yap1^F/F^* mice, hearts were isolated through a Langendorff apparatus. Freshly isolated cardiomyocytes were filtered through a 100 µm cell strainer and centrifuged at 20 × g for 5 min. The pelleted cardiomyocytes were resuspended in 1.0 ml of culture medium. Fluorescence data were collected on a BD FACS Melody cell sorter with a 100 μm nozzle. Data were further analyzed using FlowJo software.

### Luciferase reporter assay

Pgl3-basic plasmids (Promega, E1751) containing the *Mfn1* and *Dnm1l* promoters were constructed separately, named as pgl3-Mfn1 and pgl3-Dnm1. As negative controls, TEAD1-binding motifs were truncated in mutant pgl3-Mfn1 and pgl3-Dnm1 plasmids. phRL-TK was co-transfected (promega) as an internal control for normalization. To overexpress YAP1, we generated plasmid of singly overexpressed wildtype YAP1 (Yap1-wt) and phosphorylation site mutant YAP1 (Yap1-S112A). For luciferase reporter assays, 293T cells were seeded in 24-well plates overnight and then co-transfected with wild or mutant luciferase reporter vectors and other indicated plasmids according to Lipofectamine 3000 Transfection Reagent (thermofisher, L3000015) manual. Dual Luciferase Reporter Assay kit (Vazyme, DL101) was used for luciferase activity assay in each group after 48 h of post-transfection.

### Co-immunoprecipitation

293T cells were co-transfected with Tead1-Flag and Yap1-HA plasmids and cultured for 48 h. Nuero cells transfected by siNC or siVgll4 (Guangzhou RiboBio Co., Ltd.) were cultured for 48 h. Protein extracts for co-IP were prepared in Western and IP cell lysis buffer (Beyotime, P0013J). Protease inhibitor cocktail (Bimake, B14001) was added to the lysis buffer immediately before use. The protein solution incubated with pre-cleared Anti-Flag Magnetic Beads (MCE, HY-K0207), Anti-HA Magnetic Beads (MCE, HY-K0201), Dynabeads protein G (Invitrogen, 10004D), or Tead1 antibody overnight. After three washes, the immunoprecipitated proteins were eluted with 1X SDS loading buffer.

### Western blot and WES

Heart tissues or isolated cardiomyocytes were lysed in RIPA lysis buffer system (Santa Cruz Biotechnology, sc-24948) with Mini Protease Inhibitor Cocktail Tablets (cOmplete, 4693124001). Total protein concentrations were normalized using BCA analysis (Life Technologies, 23227). After boiling with 4X loading buffer for 5 min, 20 μl cell lysate of each sample was separated on a 10% gel, transferred to a PDVF membrane, and blocked by 4% BSA/TBST. Primary antibodies of aYAP1 (1:2000, Abcam, ab205270), DRP1 (1:2000, ProteinTech, 12957-1-AP), MFN1 (1:2000, Abcam, ab104274), GAPDH (1:2000, ProteinTech, 60004-1-lg), HA (1:2000, MCE, HY-K0207) and Flag (1:2000, MCE, HY-K0201) were incubated with the membrane overnight at 4 °C. After washing, membranes were incubated with HRP-conjugated secondary antibodies for 45 min. Immunoblots were then treated with Immobilon Western chemiluminescent HRP substrate (Millipore, WBKLS0500). Chemiluminescence were detected using a BioRad blot scanner. For WES Simple Western system (Bio-techne Corp., MN, USA). All the reagents used are provided in the compact kit (DM-001, DM-002, SM-W004). The primary antibody used in the study included YAP (1:20, CST, 14074S), p-YAP (Ser127) (1:20, CST, 4911S), aYAP1 (1:20, Abcam, ab205270), GFP (1:50, ProteinTech, 66002-1-Ig), and GAPDH (1:50, ProteinTech, 60004-1-lg). The secondary antibodies were ab6789 and ab6721 (Abcam). Results of WES Simple Western System were obtained using the “Lane” function of the Compass software.

### RNA-seq

Murine ventricles were flash frozen in liquid nitrogen. Two to three hearts were pooled for each biological replicate. RNA purity was checked using the NanoPhotometer^®^ spectrophotometer (IMPLEN, CA, USA). RNA concentration was measured using Qubit^®^ RNA Assay Kit in Qubit^®^ 2.0 Flurometer (Life Technologies, CA, USA). RNA integrity was assessed using the RNA Nano 6000 Assay Kit of the Bioanalyzer 2100 system (Agilent Technologies, CA, USA). Sequencing libraries were constructed using NEBNext® UltraTM RNA Library Prep Kit for total RNAs and NEBNext^®^ Multiplex Small RNA Library Prep Set for miRs for Illumina^®^ (NEB, USA) and sequenced on an Illumina HiSeq 2000 (125 nt paired end for RNA-seq). The sequencing experiments were done by Novogene Co., Ltd, Beijing, China.

Transcript abundance was determined by TopHat alignment followed by HTSeq-Count and statistical analysis by DESeq2. Gene Ontology (GO), KEGG and Reactome enrichment analysis of differentially expressed genes were implemented by the clusterProfiler R package, in which gene length bias was corrected. GO and Reactome terms with corrected P-value less than 0.05 were considered significantly enriched by differential expressed genes. GSEA was performed on expressed genes according to the software manual. Gene sets with a nominal P value of < 0.05 and an FDR of < 0.25 were considered significant. All expressed genes were Log2 or Log10 transformed, centered, and unsupervised hierarchical clustering was performed using the k-mean clustering method with Cluster 3.0 software. R software was used to visualize the clustered heatmaps. Gene sets displayed by MsigBD V7.2. Gene sets of embryonic and adult heart genes were generated according to the gene list by Uosaki et al. 2015 25.

### ATAC-seq and biochip-seq

ATAC-seq was conducted by Novogene Corp (N = 2 biological replicates per group, three hearts per replicate). Chopped frozen tissue was resuspended in homogenization buffer, and ground under homogeneous solution, followed by filtered with a cell strainer. Cell pellets were obtained by centrifugation. After iodixanol density gradient centrifugation, the nuclei band was collected form the resuspended sediment. Then, 50,000 nuclei were processed as the following ATAC-seq protocol. Briefly, nuclei were extracted from samples, and the nuclei pellet was resuspended in the Tn5 transposase reaction mix. The transposition reaction was incubated at 37 °C for 30 min. Equimolar Adapter1 and Adapter 2 were added after transposition, PCR was then performed to amplify the library. After the PCR reaction, libraries were purified with the AMPure beads and library quality was assessed with Qubit. The clustering of the index-coded samples was performed on a cBot Cluster Generation System using TruSeq PE Cluster Kit v3-cBot-HS (Illumina) according to the manufacturer's instructions. After cluster generation, the library preparations were sequenced on an Illumina Hiseq platform and 150 bp paired-end reads were generated. Nextera adaptor sequences were firstly trimmed from the reads using skewer (0.2.2). These reads were aligned to a reference genome using BWA, with standard parameters. These reads were then filtered for high quality (MAPQ ≥ 13), non-mitochondrial chromosome, and properly paired reads (longer than 18 nt). All peak calling was performed with macs2 using 'macs2 callpeak --nomodel --keepdup all -- call-summits'. For simulations of peaks called per input read, aligned and de-duplicated BAM files were used without any additional filtering.

BioChip-seq has been described previously 26. Libraries for bioChIP DNA and corresponding input samples were synthesized using KAPA Hyper Prep library kit (KK8502). Each library was generated according to manufacturer's protocol. Cycle number for adapter-ligated libraries was determined by real-time PCR prior to amplification. Libraries were sequenced (single end, 75 bp) using an Illumina NextSeq500. BioChIP-Seq libraries were aligned against the mm9 genome using Bowtie2 27. Duplicate reads and reads mapping to blacklist regions were removed. Peaks were called using MACS2 for ChIP samples with matched input samples using relaxed P-value (< 0.05). Both sequences were used to detect the TEAD1 motif in particular genes.

### Liquid chromatography-mass spectrometry (LC-MS) analysis

Heart samples were collected and extracted by ultrasonication. A Dionex Ultimate 3000 RS UHPLC system fitted with Q-Exactive quadrupole-Orbitrap mass spectrometer equipped with heated electrospqray ionization source (Thermo Fisher Scientific, Waltham, MA, USA) was used to analyze the metabolic profiling in both ESI positive and ESI negative ion modes. An ACQUITY UPLC BEH C18 column (1.7 μm, 2.1 × 100 mm) were employed in both positive and negative modes. The binary gradient elution system consisted of (A) water (containing 0.1 % formic acid, v/v) and (B) acetonitrile (containing 0.1% formic acid, v/v) and separation was achieved using the following gradient: 5-20% B over 0-2 min, 20-60% B over 2-4 min, 60-100 % B over 4-11 min, the composition was held at 100 % B for 2 min, then 13-13.5 min, 100 % to 5% B, and 13.5-14.5 min holding at 5 % B. The flow rate was 0.4 mL/min and column temperature was 45 °C. All the samples were kept at 4 °C during the analysis. The injection volume was 5 μL. The mass range was from m/z 66.7 to 1,000. The resolution was set at 70,000 for the full MS scans and 35,000 for HCD MS/MS scans. The Collision energy was set at 10, 20 and 40 eV. The mass spectrometer operated as follows: spray voltage, 3,000 V (+) and 2,500 V (-); sheath gas flow rate, 45 arbitrary units; auxiliary gas flow rate, 15 arbitrary units; capillary temperature, 350 °C.

### Statistics

The data are presented as the mean ± standard (Mean ± SD) deviation and individual data points. Two-way analysis of variance was used to assess the statistical significance of the differences between groups followed by Student-Neuman-Keuls test for multiple comparisons. P < 0.05 was considered as statistically significant difference. Statistical analysis was performed using SPSS software version 21.0 (IBM Corporation, Armonk, NY, USA). Violin plots were made with BoxPlotR 28.

## Results

### Cardiac pathological hypertrophy is accompanied by mitochondrial dysfunction

**Figure 1:**
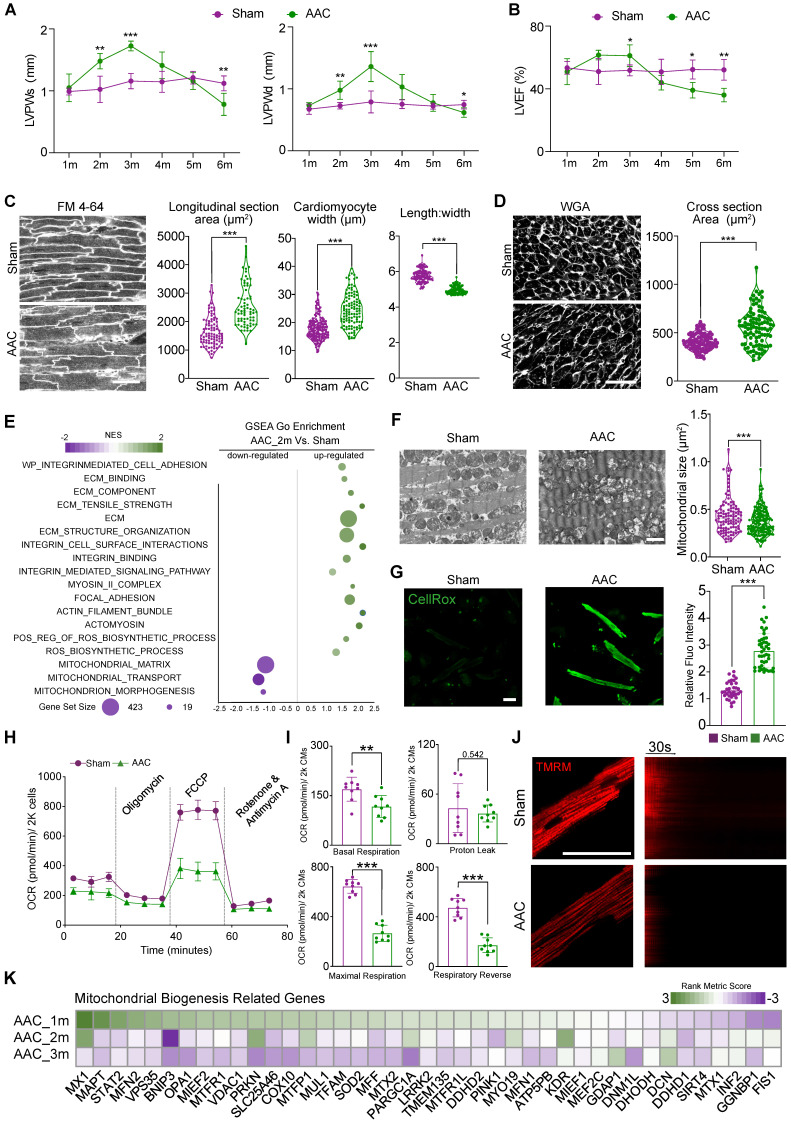
** Cardiac pathological hypertrophy is accompanied with mitochondrial dysfunction. (A-B)** The echocardiographic analysis of diastolic and systolic left ventricular posterior wall thickness (LVPWd and LVPWs) and left ventricular ejection fraction (LVEF) in sham- or AAC-treated mice. Mean ± SD. N = 12 hearts per group. **(C)** *In situ* confocal imaging of FM 4-64-stained myocardium and quantification of cardiomyocyte size and geometry. Scale bar, 50 µm. N = 3 hearts per group **(D)** WGA staining of myocardial cryosections and quantification of cardiomyocyte cross section area. Scale bar, 50 µm. N = 3 hearts per group. **(E)** Scatter plot displaying the differentiated GO term enrichments in AAC hearts of RNA-seq at 2 months. N = 3 hearts per group. **(F)** Transmission electron microscopic (TEM) images of sham and AAC heart tissues and the quantification of mitochondrial section area. Scale bar, 1 µm. N = 6 hearts per group. **(G)** CellROX staining for adult isolated cardiomyocytes indicated a significant increasing of fluoresces density of CellROX among AAC mice by qualitative analyses. N = 3 hearts per group. **(H-I)** Measurement of the oxygen consumption rates (OCRs) of cardiomyocytes isolated from Sham and AAC mice. OCRs were normalized by total cardiomyocyte number per group. N = 9 hearts per group. **(J)** TMRM staining for isolated cardiomyocytes from AAC and sham hearts. Line scanning for 3 mins had been used to record the resist of mitochondrial membranous potentials. N = 3 hearts per group. **(K)** Heatmap of mitochondrial biogenesis related genes based on RNA-seq are shown among three different time points post AAC vs sham ones, indicating the impairment of mitochondrial biogenesis upon mechanical stress overload. N = 3 for each group. Mean ± SD. Student's t-test or Mann-Whitney U-test was applied. **P < 0.01. ***P < 0.001. Non-significant P values in parenthesis.

We performed AAC surgery on 1-month old mice to induce pressure overload to the heart. Compared with the more commonly used TAC model, AAC induces chronic and progressive cardiac hypertrophy that better mimics the pathogenic process in patients with hypertensive heart diseases. Thus, AAC represents an optimal model of HFpEF for studying 16, 29. In our AAC model, the ventricular wall thickness appeared normal 1 month after surgery, but increased by months 2 and 3 (Figure 1A). Cardiac fraction shortening stayed normal by the third month after AAC surgery with presentative (Figure S1A), while left ventricular ejection fraction (LVEF) slightly increased at the end of months 3 (Figure 1B). HE staining demonstrated myocardial hypertrophy in left ventricle (Figure S1B). Besides, increased interstitial fibrosis (Figure S1C-D) and the upregulation of cardiac stress markers (Figure S1E) such as *Nppa* were detectable in 3 months AAC-treated ventricles, which indicated HFpEF. Four months after AAC, we began to observe a reduction in cardiac contractile function and the thickness of ventricular walls. Moreover, we found a significant reduction in both the morphologic and functional changes associated with LVPW thickness and LVEF or FS, which reached the status of HFrEF in the following months 5 and 6 (Figure 1A-B). These data showed that the first three months after AAC surgery provided an ideal time window to study the mechanism underlying cardiac pathological hypertrophy under chronic pressure overload with persevered ejection fraction.

At the cellular level, we next performed *in situ* myocardium imaging using FM 4-64 as a cell boundary dye 30 to measure cardiomyocyte size and shape changes 3 months after AAC. We measured cardiomyocyte cross-sectional area in sections stained by wheat germ agglutinin (WGA). Both analyses confirmed that AAC triggered cardiac hypertrophy mainly through an increase in cardiomyocyte width (Figure 1C-D).

To further analyze biological changes affecting cardiomyocytes after AAC, RNA-seq was performed on heart tissues at 2 months post-AAC and on control heart tissues (N = 3 biological replicates per group, three hearts per replicate). Scatter plots demonstrated that the upregulated GSEA terms were involved in extracellular matrix (ECM) remodeling, integrin activation, the myosin II complex, focal adhesion, and actomyosin, which formed an ECM-Integrin-MYOSIN mechanotransduction signaling pathway. Furthermore, significantly elevated ROS-accumulation-related terms were identified, consistent with the downregulation of mitochondrial morphogenesis- and structure-related terms after mice were subjected to AAC (Figure 1E). We next performed TEM analysis to examine the mitochondria 3 months after AAC treatment. We found that the cross-sectional area of mitochondria significantly decreased after AAC treatment, which indicated the activation of mitochondrial hyperfragmentation (Figure 1F). Mitochondrial cristae also appeared disorganized in the AAC group, compared to sham animals (Figure 1F). Enhanced CellROX staining was observed in isolated cardiomyocytes belonging to the AAC group, indicating that ROS production was increased (Figure 1G). We measured the OCRs of cardiomyocytes that were isolated from sham- or AAC-treated mice and observed a significant decrease in the oxidative respiration of cardiomyocytes in the AAC group (Figure 1H-I). The cardiomyocytes of mice in the AAC group also exhibited reduced TMRM staining (Figure 1J), which represented decreased mitochondrial membrane potentials 19. The GO term: mitochondrial biogenesis was selected to present on the heatmap of the rank metric scores from GSEA (Figure 1K). Together, these data indicated that the induction of cardiac pathological hypertrophy with HFpEF was tightly associated with the perturbation of mitochondrial ultrastructure and function.

### YAP1 activation is responsible for mitochondrial perturbation and cardiomyocyte hypertrophy

**Figure 2:**
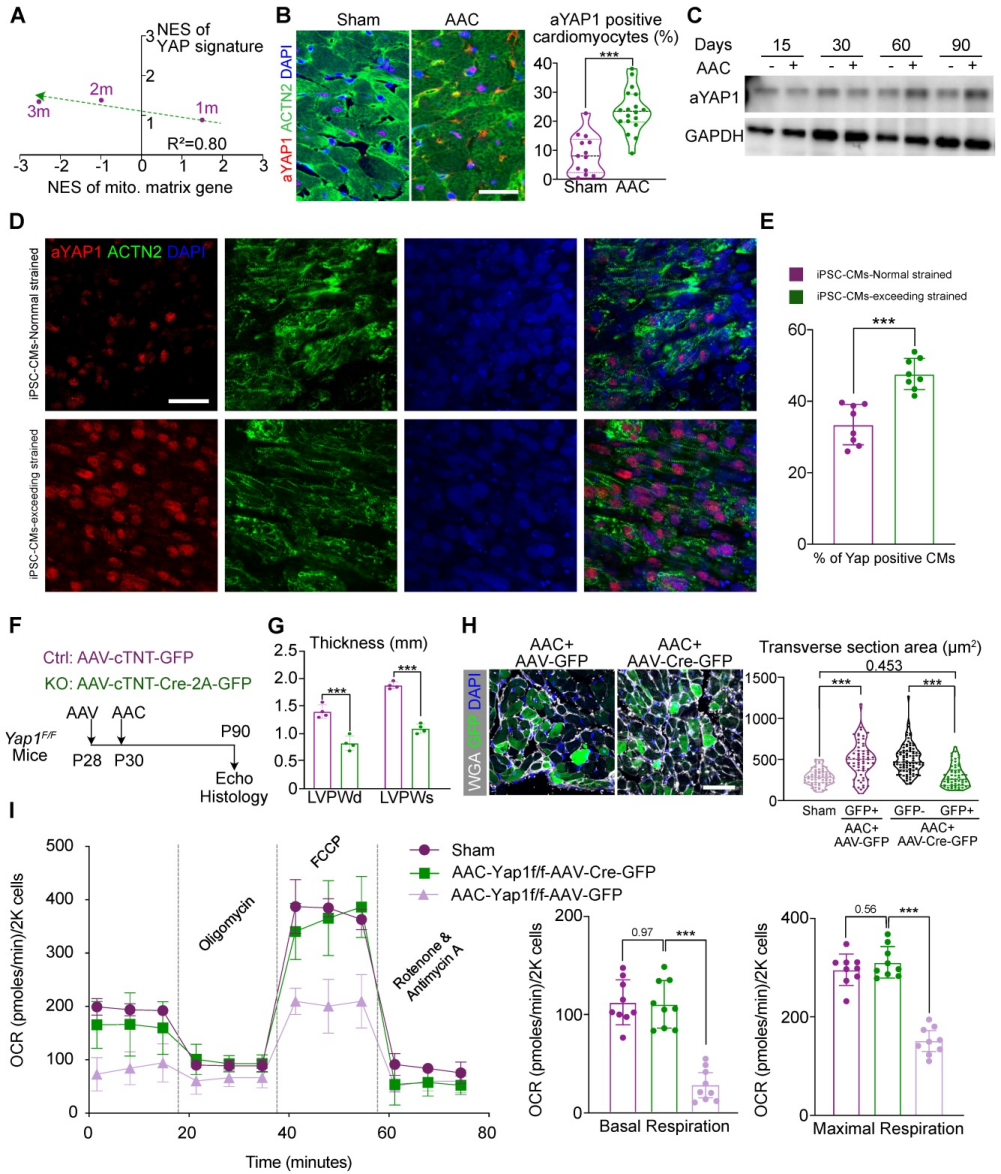
** YAP1 activation is responsible for mitochondrial perturbation and cardiomyocyte hypertrophy. (A)** Normalized enrichment scores (NESs) of gene sets related to YAP signaling and mitochondria. At 1-, 2-, and 3-month post AAC surgery, ventricular tissues were subjected to RNA-seq and gene set enrichment analysis (GSEA). N = 3 for each group. **(B)** Immunofluorescence staining for activated YAP (aYAP) in cardiac tissue sections and quantification of aYAP positive cardiomyocytes. Scale bar, 50 µm. N = 6 hearts per group. **(C)** Immunoblotting analysis for aYAP in cardiac tissues. N = 3 hearts per group. **(D)** Immunostaining for YAP and ACTN2 on iPSC derived cardiomyocytes tissue bundles which are subjected to normal and exceeded stretch. **(E)** The quantitative result of the ratio of YAP+ cardiomyocytes on iPSC derived tissue bundles. N = 8, each dot presents the average results of 5 fields/slice. Bar, 50 µm. **(F).** Experimental design to determine the role of YAP1 in AAC-induced cardiac responses. **(G)** Echocardiographic measurement of ventricular wall thickness in AAC-treated *Yap1^F/F^* mice. Mean ± SD. N = 4 hearts per group. **(H)** WGA-stained cardiac cryosections and quantification of cardiomyocyte transverse section area. In AAC and AAV treated hearts, GFP+ and GFP- cardiomyocytes were separately measured. Scale bar, 50 µm. N = 3 hearts per group. **(I)** Measurement of the oxygen consumption rates (OCRs) of isolated GFP positive cardiomyocytes after FACS sorting. OCRs were normalized by total cardiomyocyte number per group. N = 9 hearts per group. Mean ± SD. In statistical analysis, student's t-test or Mann-Whitney U-test was applied. **P < 0.01. ***P < 0.001.

A time-series RNA-seq analysis had been involved to understand the transcriptional changes at 1, 2, and 3 months after AAC induction. In the 2-month and 3-month data, GSEA revealed a significant decrease in the expression of adult-specific cardiac genes and an increase in the expression of fetal cardiac genes, which confirmed the well-established phenotypes for cardiac pathogenesis (Figure S2A) 31. In addition, we observed mitochondria-related gene ontologies as the most down-regulated sets and cell adhesion and ECM gene ontologies as the major up-regulated sets. Strikingly, at 1 month post AAC, the expression of many of these gene sets (e.g., genes related to lipid oxidation), was reversed (Figure S2B), suggesting that a critical transcriptional switch occurred in the early phase of AAC-induced hypertrophy. YAP signaling [Gene Set: CORDENONSI_YAP_CONSERVED_SIGNATURE] was revealed to be a major gene set that was activated by AAC, and was negatively corelated with the transcriptional switch of mitochondria-related gene sets (Figure 2A and Figure S2C).

To validate the activation of YAP signaling, we applied an antibody that selectively recognized non-phosphorylated, activated YAP1 proteins (aYAP1) 32 to perform immunofluorescence and western blot analysis on sham- and AAC-treated ventricle tissues. The aYAP1 antibody is designed to selectively target non-phosphorylated YAP proteins alone. As previous reports, this selective antibody was validated by a series studies since this product released in recent 5 years 33-36. In our study, we also performed total YAP and p-YAP immunoblotting to validate the specificity and accuracy of the aYAP1 antibody. These experiments showed that the ratio of aYAP1-positive cardiomyocytes (Figure 2B) and the amount of aYAP1-bound proteins (Figure 2C) were both increased by the second month after AAC surgery. Besides, we also found the ratio of aYAP1-positive non-cardiomyocyte was increased by 2 months after AAC (Figure S2D). Next, we labelled cardiomyocytes by injecting mice with a dose of AAV-cTNT-GFP (4×10^10^ vg/g) at P28, prior to performing AAC and sham operation at P30. The cardiomyocytes were then isolated at 2 months post AAC and the labeled GFP-positive cardiomyocytes were sorted by flow cytometry. This pure population of cardiomyocytes were used to measure the activation of YAP, as it excluded non-specific signals from non-cardiomyocytes. WES was used to measure the levels of YAP, p-YAP, and aYAP1, which were consist with the results of aYAP1 immunoblotting (Figure S2E). To further validate the activation of YAP1 under conditions of excessive mechanical stress, WTC iPSC cell lines were differentiated into cardiomyocytes following the standard protocol reported previously 22. A pure, mature population of cardiomyocytes was then compacted into bundle tissue and subjected to 25% stretching (i.e., normal stress) or 50% stretching (i.e., excessive stress), followed by immunostaining for aYAP1. More aYAP1 positive cardiomyocytes were recorded in the samples subjected to excessive stress (Figure 2D-E). Collectively, these experiments demonstrated that YAP1 is activated after exposure to chronic mechanical stress.

Next, we aimed to determine if there was a causal relationship between YAP1 activation and the AAC-induced cardiac phenotypes. Thus, we administered a low-dose of AAV vector (2.0×10^10^ vg/g) expressing Cre-2A-GFP specifically into the cardiomyocytes of *Yap1^F/F^* mice 37 to deplete YAP1. We then examined the impact of YAP1 depletion on AAC-induced cardiac hypertrophy. The AAV vector expressing GFP alone was used as a control (Figure 2F). Immunoblotting demonstrated the significant attenuation of aYAP1 after AAV-cTNT-Cre-2A-GFP application. The dominant mitochondrial biogenesis-associated factors, DRP1 and MFN1, were activated after YAP1 knock-out, which was exact opposite of our AAC RNA-seq results (Figure S3A). We isolated the cardiomyocytes both from the AAV-cTNT-Cre-2A-GFP- and AAV-CTNT-GFP-transduced AAC mice by FACS sorting. Around 25% of the cardiomyocytes were GFP-positive in both the treated and control groups (Figure S3B). In *Yap1*-silenced hearts, AAC-induced ventricular wall thickening was significantly attenuated (Figure 2G), while both the LVEF and FS remained (Figure S3C). We used the GFP marker to label the YAP1-depleted cardiomyocytes, and found that these cells appeared smaller than their adjacent non-transduced cardiomyocytes (Figure 2H). OCR measurement of sorted GFP-positive isolated cardiomyocytes demonstrated that YAP1 depletion was sufficient to restore mitochondrial functions in the hearts of AAC mice (Figure 2I). Together, these results showed that YAP1 activation is a major factor that induces pathological hypertrophy and mitochondrial dysfunction in murine cardiomyocytes post-AAC.

### YAP1 activation is sufficient to trigger mitochondrial dysfunction and pathological hypertrophy

**Figure 3:**
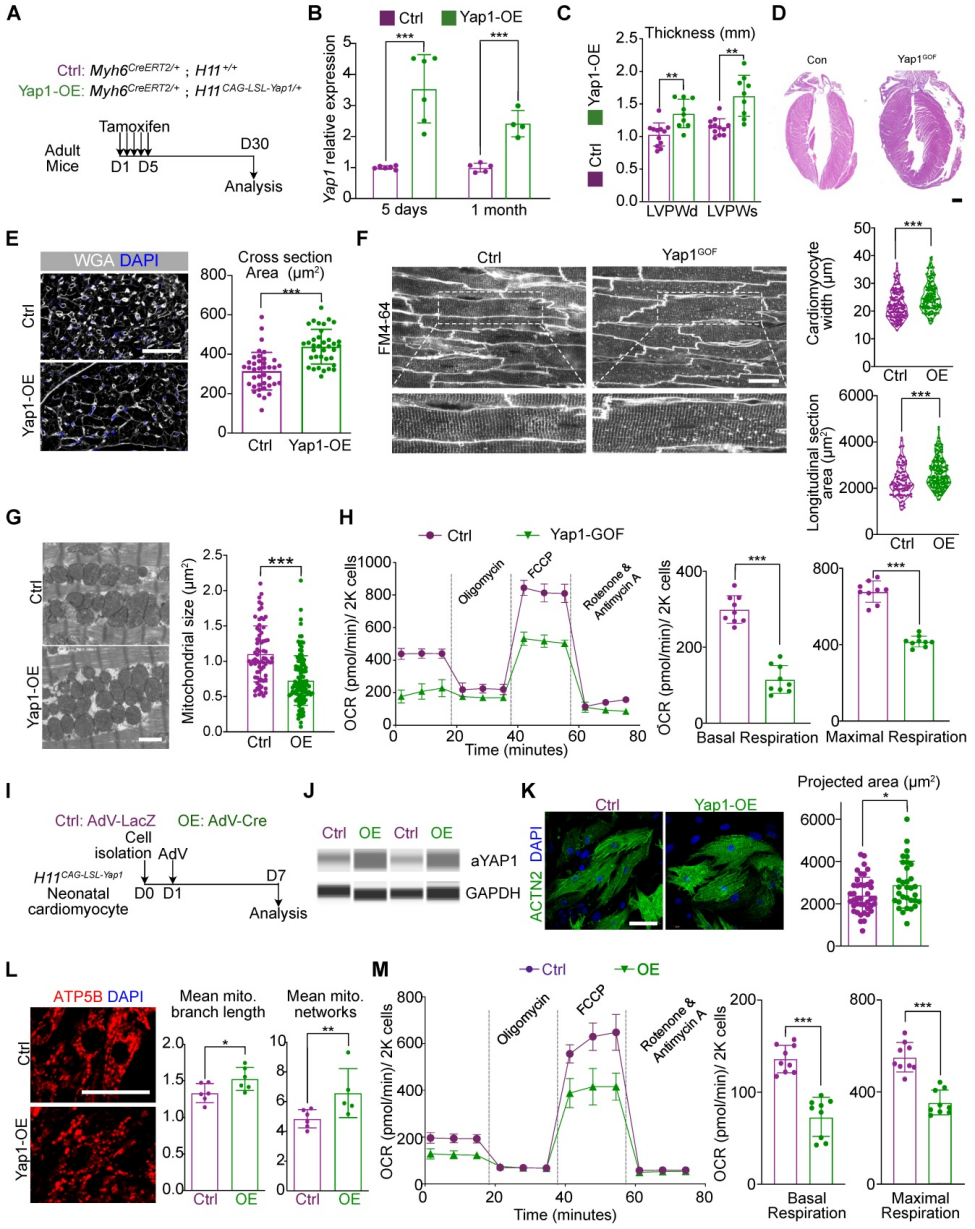
** YAP activation is sufficient to trigger mitochondrial dysfunction and pathological hypertrophy. (A)** Experimental design for YAP1 overexpression *in vivo*. Tamoxifen was injected intraperitoneally to induce YAP1 overexpression specifically in cardiomyocytes. **(B)** qPCR showed the significant increased expression of activated YAP in cardiac tissue. N = 6 hearts per group. **(C)** Echocardiographic measurement of LVPW thickness. N = 9 hearts per group. **(D)** HE staining demonstrated significant hypertrophy on YAP1-overexpression heart. N = 3 hearts per group. **(E)** WGA staining of heart tissues and quantification of cardiomyocyte cross section area. N = 3 hearts per group. **(F)** *In situ* imaging had been used to present the hypertrophic CM in in YAP1-overexpression mice. Quantitative analyses showed the increased cell size and length of CM width. N = 3 hearts per group. **(G)** TEM analysis and quantification of mitochondria cross section area. N = 6 hearts per group. **(H)** OCR measurement of isolated cardiomyocytes normalized to cell number. Mean ± SD. **(I)** Experimental design for YAP1 overexpression *in vitro*. Primary neonatal murine ventricular cardiomyocytes (NMVMs) were isolated at P0, incubated with AdV on day 1, and analyzed on day 7. **(J)** WES simple protein measurement showed the increased expression of aYAP. N = 3 hearts per group. **(K)** ACTN2 staining and measurement of projected cell area of NMVMs. N = 3 biological replicates in each group. **(L)** ATP5B staining and quantification of mitochondria morphology in NMVMs. N = 3 biological replicates in each group. **(M)** OCR measurement of NMVMs normalized to cell number. N = 9 hearts per group. Mean ± SD. Student's t-test or Mann-Whitney U-test was applied. *P < 0.05. **P < 0.01. ***P < 0.001.

To further assess the impact of YAP1 activation on cardiac hypertrophy, we created mice carrying *Myh6^CreERT2^* and *H11^CAG-LSL-YAP1^* alleles. In this model, tamoxifen injection would activates CreERT2 specifically in cardiomyocytes, which recombines with the loxp-Stop-loxp (LSL) cassette in the *H11^CAG-LSL-YAP1^* allele and activates *Yap1* overexpression (Figure 3A). Using this method, a 3-4 fold increase in YAP1 expression was detected at the mRNA and protein levels (Figure 3B and Figure S4A). In addition, a reduction in DRP1 and MFN1 expression was also observed by immunoblotting by 1 month after *Yap1* activation (Figure S4A).

**Figure 4:**
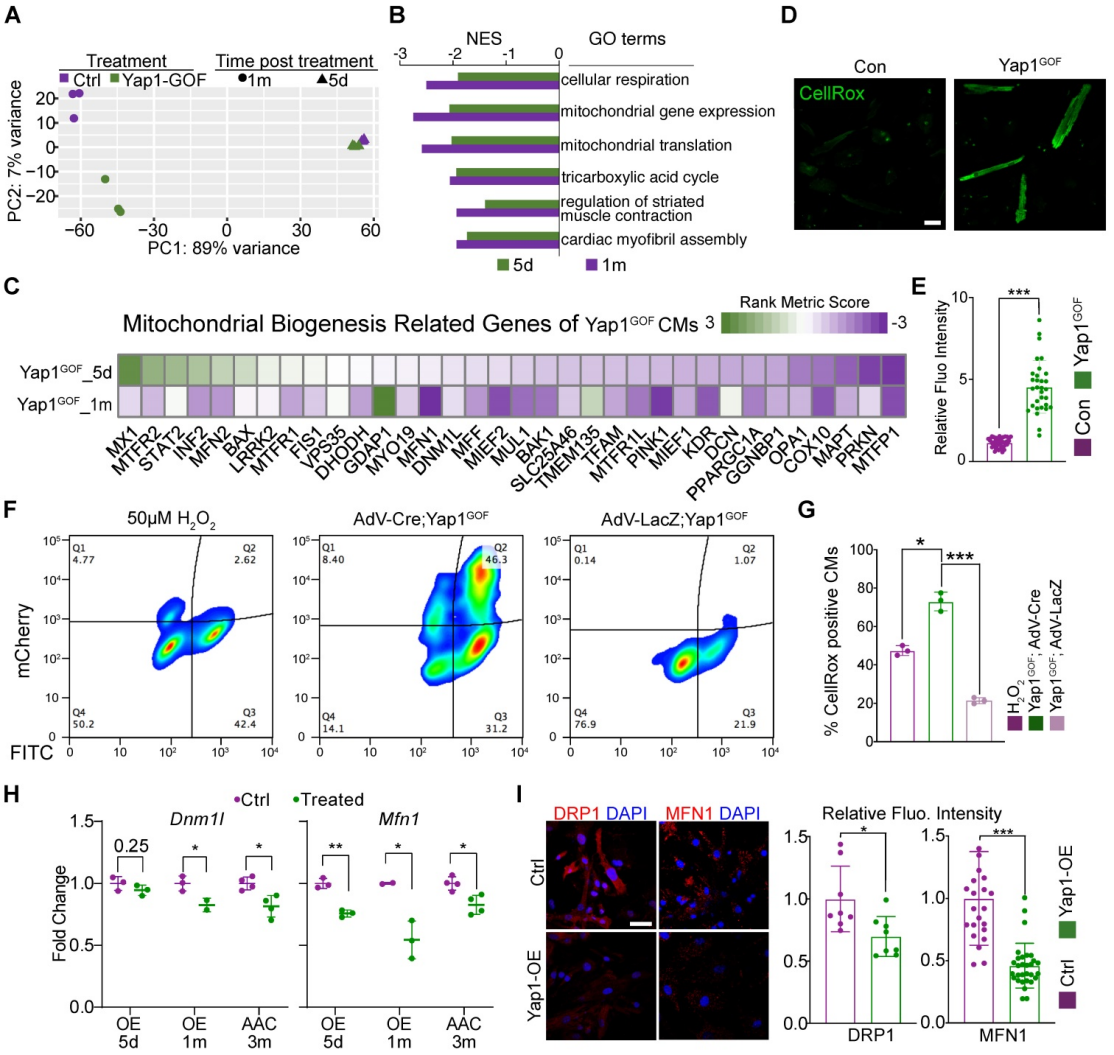
** YAP1 overexpression triggers cardiac hypertrophy by impairing mitochondrial gene *Dnm1l* and *Mfn1*. (A)** Principal component analysis (PCA) of RNA-seq data for control and YAP1 overexpressing hearts treated for 5 days or 1 month. N = 3 hearts per group. **(B)** GSEA of mitotic nuclear division gene set in the YAP1 overexpression RNA-seq analysis. N = 3 hearts per group. **(C)** Heatmap of mitochondrial biogenesis related genes based on RNA-seq are shown among three different time points post YAP1 activation, indicating the impairment of mitochondrial biogenesis upon YAP1 activation. N = 3 for each group. **(D-E)** CellROX staining for adult isolated cardiomyocytes indicated a significant increasing of fluoresces density of CellROX among AAC mice by qualitative analyses. N = 3 biological replicates in each group. **(F)** Cultured YAP1-overexpression NMVMs were treated with H_2_O_2_ (positive control), AdV-LacZ, and AdV-Cre. The fraction of CellROX positive cells was measured by FACS. N = 3 biological replicates in each group. **(G)** Quantification of CellROX positive cells between H_2_O_2_, AdV-LacZ and AdV-Cre treated groups. Since LacZ control cells did not activate the mcherry reporter, we compared total CellROX positive cells among groups. **(H-J)** *Dnm1l and Mfn1* were measured by quantitative PCR and immunofluorescent staining. *Dnm1l* and *Mfn1* had markedly decreased upon YAP1 activation both in heart tissues and cultured NMVMs**.** Scale bar, 50 µm. N = 3 biological replicates in each group. Mean ± SD. Student's t-test was applied. *P < 0.05. ***P < 0.001. Non-significant P values in parenthesis.

We subsequently performed echocardiographic and histological analyses and observed an increase in the ventricular wall thickness in the hearts of mice overexpressing YAP1 (Figure 3C-D). At the same time, the LVEF was slightly increased by 1 month after YAP1 activation, while FS remained unchanged (Figure 4B), which was similar to the echocardiographic parameters of mice at 3 months post-AAC. We also detected the upregulation of fibrosis and cardiac stress markers (Figure S4C) such as *Ctgf*, *Nppa, Nppb* in YAP1-overexpressing ventricles. Moreover, hyper-fibrosis was confirmed by Masson straining with increased quantification of fibrotic area (Figure S4D), which indicated a similar HFpEF to that of AAC mice. Under the conditions, the mitochondrial DNA transcription was also inhibited (Figure S4C). Myocardial hypertrophy was further validated by the increased cardiomyocyte cross-sectional area in the YAP1-overexpressing myocardial sections (Figure 3E) and *in situ* optical sections analyzed by confocal microscopy (Figure 3F). TEM analysis showed that YAP1-overexpressing cardiomyocytes had a smaller mitochondrial cross-sectional area, indicating mitochondrial hyper-fragmentation. (Figure 3G). OCR measurement demonstrated a reduction in cardiomyocyte respiration after YAP1 overexpression (Figure 3H). Interestingly, we failed to identify any evidence on induced proliferation with ki67- or pH3-positive cardiomyocytes after 1 month of YAP-overexpression (Figure S4E).

To determine the mechanism, we next isolated primary neonatal murine ventricular cardiomyocytes (NMVMs) carrying the* H11^CAG-LSL-YAP1^* alleles and transduced these cells with AdVs to express Cre recombinase 19 and induce YAP1 overexpression *in vitro* (Figure 3I). Western blot analysis confirmed the robust overexpression of aYAP1 upon AdV-Cre transduction, using AdV-LacZ as a control vector (Figure 3J). Analysis of ACTN2-positive NMVMs projected cell area showed that YAP1 was sufficient to induce cardiomyocyte hypertrophy *in vitro* (Figure 3K). We probed ATP5B as a marker to study mitochondrial morphology and found that YAP1 overexpression remodeled the mitochondria, causing them to adopt a more filamentous and branched network, as quantified by the MiNA with Fiji software 24 (Figure 3L). YAP1-overexpressing NMVMs also exhibited reduced oxidative respiration (Figure 3M). Thus, YAP1 hyperactivation is sufficient to trigger cardiomyocyte hypertrophy while impairing mitochondrial function.

### Mitochondria dysfunction is responsible for YAP1-induced cardiac hypertrophy

We next performed RNA-seq analysis of cardiac tissues that overexpressed YAP1 for 5 days or 1 month to study the impact of YAP1 overexpression on gene expression. GSEA demonstrated that the activation of YAP1 was associated with a conserved signature by 1 month of induction (Figure S5A). Principle components analysis (PCA) showed that at 5 days after induction, YAP1-overexpression data already separated from the control data, which was even more pronounced after 1 month (Figure 4A). Venn diagrams showed the overlap between the differentially expressed genes at the two time points after YAP1 activation and TEAD1 motif-verified genes (Figure S5B). A total of 159 overlapping genes were identified and GO terms enrichment analysis indicated that these genes were involved in mitochondrial-related processes, such as mitochondrial morphogenesis and functional maintenance (Figure S5C). The GO term 'mitochondrial inner membrane' was selected to present on the heatmap of data from AAC and YAP1-overexpressing mice as it produced a large overlap between gene expression features (Figure S5D).

GSEA showed that the major gene sets that were differently regulated at 5 days versus 1 month of YAP1 overexpression (Figure S5E). By contrast, mitochondrial and metabolic genes were the most downregulated gene expression programs at both time points (Figure 4B). Thus, the pattern of YAP1-triggered gene expression changes depended on the duration of YAP1 activation. A heatmap was generated to show the expression of genes related to mitochondrial biogenesis (Figure 4C). We analyzed the ROS levels of isolated YAP1-overexpressing cardiomyocytes. CellROX staining results showed an increased in the ROS levels after YAP1 activation (Figure 4D-E). Besides, NMVMs were also stained with CellROX and measured by flow cytometry. As expected, the positive control cells treated with 50 µM H_2_O_2_ exhibited elevated CellROX fluorescence (Figure 4F, left panel). In AdV-LacZ-treated NMVMs, most cells had a low CellROX signal, consistent with low ROS levels (Figure. 4F, right panel). In contrast, mCherry positive, AdV-Cre-transduced YAP1-overexpressing cardiomyocytes exhibited markedly increased CellROX fluorescence (Figure 4F, middle panel). Quantitative analysis across replicates confirmed that YAP activation markedly increased cardiomyocyte ROS production (Figure 4G). As mitochondrial biogenesis had been impaired by YAP activation, *Dnm1l* and *Mfn1* were measured by qPCR and immunostaining. *Dnm1l* and *Mfn2* had markedly decreased expression upon YAP activation both in heart tissues and in cultured NMVMs (Figure 4H-I). The inhibition of *Mfn2* was also measured by quantitative PCR and immunostaining (Figure S6A-B).

VGLL4 is an inhibitor for YAP. We therefore used siRNA to knock-down *Vgll4* in primary cultured wild type cardiomyocytes to validate the results of YAP1 overexpression, as an alternative way of inducing YAP activation. Once the inhibition of *Vgll4* expression was confirmed by qPCR (Figure S7A), we used Co-IP to measure the interactions between YAP1, VGLL4, and TEAD1. We found that the inhibition of *Vgll4* increased the activation of YAP1 and its binding to TEAD1 (Figure S7B). GSEA demonstrated the activation of YAP signature was associated with decreased mtDNA transcription. Besides, leading edge analyses also revealed that early heart development genes were positively enriched and late heart development genes were negatively enriched (Figure S7C). Heatmap presented similar view of mitochondrial biogenesis genes expression, indicating the impairment of mitochondrial biogenesis (Figure S7D). Scatter plots demonstrated that the accumulation of ROS and mitochondrial dysfunction were similar to the results from YAP1-overexpressing NMVMs (Figure S7E). Immunostaining for aYAP1 confirmed that activation of YAP post *Vgll4* inhibition (Figure S8A-B). MitoTracker staining also demonstrated the hyper-fragments changes of mitochondria (Figure 8C-D).

### *Dnm1l* and *Mfn1* are direct targets of TEAD1-YAP

**Figure 5:**
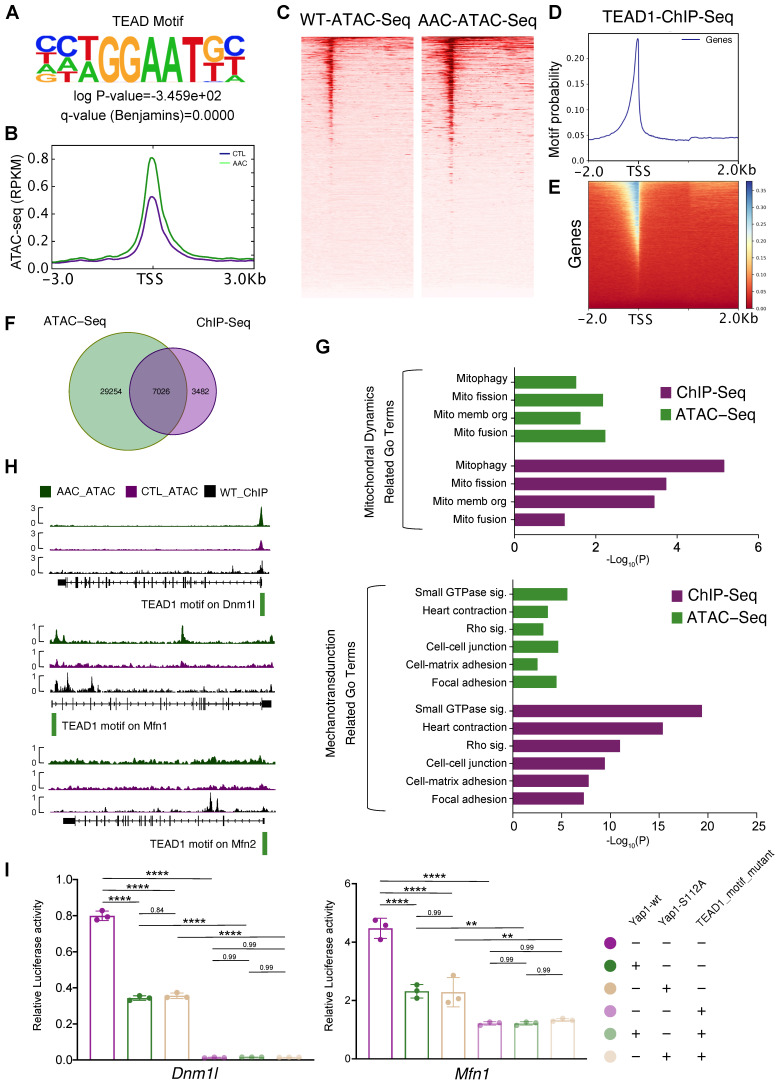
** Dnm1l and Mfn1 are direct targets of TEAD1-YAP. (A)** Unbiased motif discovery with mostly accessible ATAC-seq peaks showed that TEAD motif significantly enriched. N = 3 hearts per group. **(B-C)** Increased accessibility at TEAD element by ATAC-seq signals peaks at gene body and TEAD motif peaks. N = 3 hearts per group. **(D-E)** BioChIP-seq signals had been explored to identify the binding sites in genome. N = 3 hearts per group. **(F)** Venn plots to show the overlap of targeted TEAD1 motif from ATAC-seq and bioChIP-seq and identified 7,026 binding sites in cardiomyocytes. N = 3 hearts per group. **(G)** GO terms enrichment had been done based on the 7,026 bind sites located genes. Both ATAC-seq and bioChIP-seq peaks were markedly enriched among mitochondrial related genes, and less enriched in mechanotransduction related terms. N = 3 hearts per group. **(H)** TEAD1 occupancies had been identified near *Dnm1l* and *Mfn1* promoters, and weak occupancy could be targeted at *Mfn2* promoter. N = 3 hearts per group. **I.** The luciferase assessments of Yap1-wt and Yap1-S112A on *Dnm1l/Mfn1*-WT or *Dnm1l/Mfn1*-MUT vectors. N = 3 biological replicates in each group. Student's t-test applied. **P < 0.01. ****P < 0.0001. Non-significant P values in parenthesis.

To determine the mechanisms by which *Yap1* regulated mitochondrial homeostasis in cardiomyocytes, the transcriptional role of YAP1 needed to be analyzed. However, YAP1 functions as a transcription co-factor and does not bind to DNA directly. Thus it was difficult to pull-down YAP1 with its DNA-binding sequence by ChIP, and a stable system of ChIP-seq for YAP1 could not be established during the course of the current study (both in our and William T. Pu's laboratories). Therefore we concluded that ChIP-seq for YAP1 would not be reliable. Instead, we adopted an alternative approach was taken by generating a knock-in allele of *Tead1* fused to a biotin acceptor peptide (BIO tag) at the carboxyl-terminus (*Tead1^fbio^*). BIO is specifically biotinylated by the *Escherichia coli* biotin ligase BirA, which was expressed using the Rosa26^BirA^ allele 26. This highly sensitive system circumvented the caveats of antibody-based ChIP by permitting the exploration of TEAD1 binding sites. We showed that TEAD1 was predominantly binding to YAP1 as a transcription factor. Unbiased motif discovery with mostly accessible ATAC-seq peaks showed that the TEAD motif was significantly enriched in mice at 2 months post-AAC (Figure 5A). Increased accessibility at the TEAD element were also revealed by ATAC-seq signal peaks at the gene body (Figure 5B) and TEAD motif peaks (Figure 5C). Tead1^fbio^ mice were then crossed with Rosa26^BirA^ mice to generate the bioChIP system for TEAD1, which has been described previously by Akerberg et al 26. bioChIP-seq signals were explored to identify the binding sites for TEAD1 in the genome (Figure 5D-E). Venn diagrams were used to show the overlap between targeted TEAD1 motif data from ATAC-seq and bioChIP-seq; we identified 7,026 binding sites in cardiomyocytes (Figure 5F). GO term enrichment analyses were conducted based on the genes containing the 7,026 binding sites. Both ATAC-seq and bioChIP-seq enriched TEAD1 peaks were markedly enriched among mitochondrial-related genes and mechanotransduction-related terms (Figure 5G). Further characterization of the properties of TEAD1 peaks included analysis of the location of TEAD1 sites with respect to transcription start sites (TSSs), which showed that almost 40% of mitochondrial elements were proximal to promoters. Compared with other elements, mitochondrial elements were more proximal to promoters (TSS ± 1000 bp) which indicated that the TEAD1-YAP1 complex regulated mitochondrial-related genes. The above results were indicative of the disruption of mitochondrial biogenesis and the induction of mitochondrial hyperfragmentation, which were the dominant changes observed upon chronic mechanical overloading and YAP1 overexpression. Moreover, a previous study had identified that an imbalance between mitochondrial fusion and fission would lead to mitochondrial biogenesis impairment and hyperfragmentation, which were guided by *Dnm1l, Mfn1 and Mfn2* 38. Subsequently, the roles of these major regulating genes in mitochondrial biogenesis (*Dnm1l*, *Mfn1,* and *Mfn2*) was investigated for whether they were direct targets of the TEAD1-YAP1 complex, as all of these genes were significantly inhibited by AAC and YAP1 overexpression. Strong TEAD1 occupancies were identified near the *Dnm1l* and *Mfn1* promoters (Figure 5H, upper and middle panels), and a weak occupancy could be detected at the *Mfn2* promoter (Figure 5H, lower panel).

Having confirmed the targets of the TEAD1-YAP1 complex on *Dnm1l*, *Mfn1*, and *Mfn2*, which regulate mitochondrial biogenesis, the mechanism of TEAD1-YAP1 complex regulation was explored. In the luciferase reporter assay, *Tead1* overexpression induced the luciferase activities of pgl3-Dnm1l and pgl3-Mfn1 groups (Figure S9A, left and middle panel) and inhibited the luciferase activity of the pgl3-Mfn2 group (Figure S9A, right panel). The TEAD1 motif mutant pgl3-Dnm1l vector had a low level of luciferase activity, while the overexpression of TEAD1 did not induce the luciferase activity of the TEAD1 motif mutant pgl3-Dnm1l vector (Figure S9A, left panel), indicating that the transcription of *Dnm1l* was regulated entirely by the TEAD1 complex. In addition, reduced luciferase activity was observed following expression of the TEAD1 motif mutant pgl3-Mfn1 vector, while the overexpression of TEAD1 could enhance the luciferase activity of the TEAD1 motif mutant pgl3-Mfn1vector (Figure S9A, middle panel), indicating that the TEAD1 complex partially regulated *Mfn1*. However, the luciferase activity of TEAD1 motif mutant pgl3-Mfn2 vector increased, indicating that TEAD1 would inhibit the transcription of *Mfn2* (Figure S9A, right panel). Based on the luciferase experiments and TEAD1 motif peak enrichment, *Dnm1l* and *Mfn1* were revealed to be regulated by TEAD1 complex in same manner, while *Mfn2* was considered to be regulated independently of TEAD1-YAP in AAC and YAP1-overexpressing mice. Moreover, the singly overexpressed wildtype *Yap1* (Yap1-wt) and the phosphorylation site mutant *Yap1* (Yap1-S112A) could inhibit the luciferase activities of both the pgl3-Dnm1l and pgl3-Mfn1 vectors (Figure 5I). In contrast, the overexpression of Yap1-wt and Yap1-S112A did not alter the luciferase activities of Dnm1l and Mfn1 upon TEAD1 motif mutant expression (Figure 5I).

Accordingly, *Dnm1l* and *Mfn1* are the direct targets for the TEAD1-YAP1 complex. Moreover, the activation of YAP1 inhibits the expression of *Dnm1l* and *Mfn1*, thereby impairing mitochondrial biogenesis.

### Rescue of mitochondrial morphology and function by re-expression of *Dnm1l* and *Mfn1*

To determine the potential connection between DRP1, MFN1 and YAP1-induced cardiomyocyte hypertrophy, we next examined if the knockdown of DRP1 and MFN1 was sufficient to induce cardiomyocyte hypertrophy. We transfected siRNAs targeting *Dnm1l* and *Mfn1* into NMVMs and observed significant decrease in the levels of targeted mRNAs (Figure S10A). This did not alter the YAP signaling expression (as determined by GSEA based on RNA-seq analyses), indicating that DRP1 and MFN1 were the downstream molecules of YAP signaling (Figure S10B). Analysis of the ACTN2-postive cardiomyocyte projected area revealed an increase in cell size after the combined depletion of DRP1 and MFN1 together (Figure S10C). Furthermore, siDnm1l+MFN1 cardiomyocytes were negatively enriched in the GO term of mitochondrial translation and positively enriched in early heart development genes, which was indicative of myocardial remodeling (Figure S11A). Scatter plots revealed a similar GO terms enrichment of siDRP1+MFN1 in the direction of upregulated and downregulated genes, compared with cardiomyocytes exposed to short-term (5 days) YAP1 activation (Figure S11B). Several of the enriched GO terms were connected to ROS metabolism and ROS-related damage.

**Figure 6:**
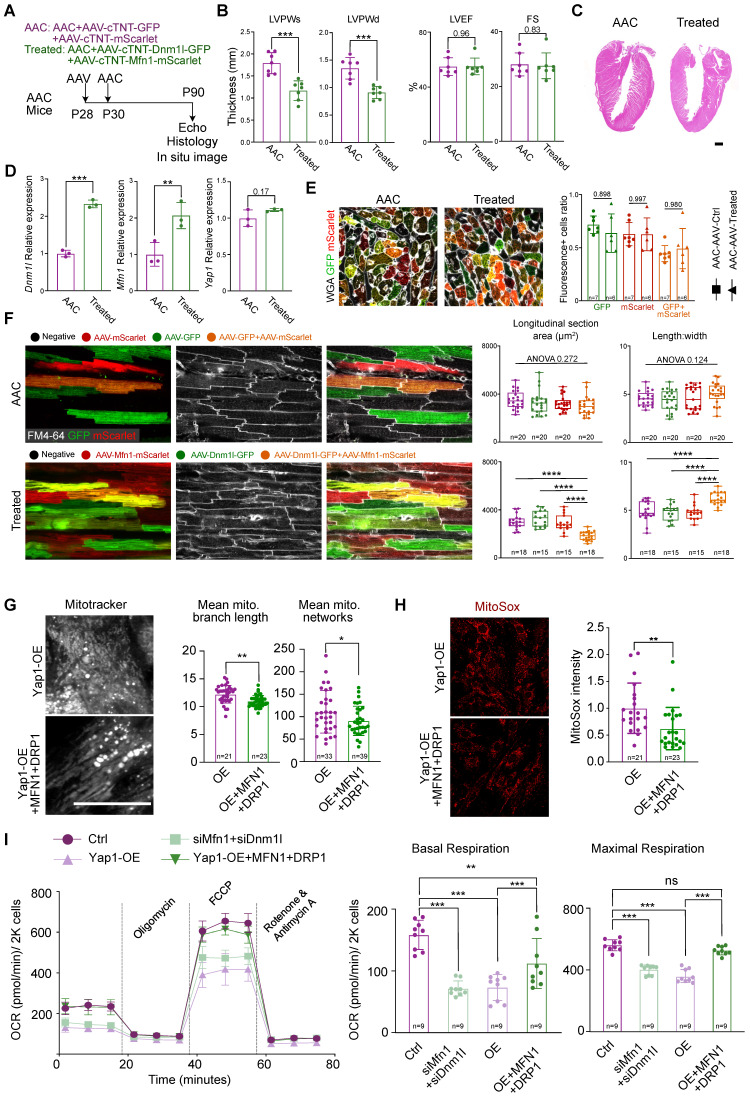
** Rescue cardiac hypertrophy by re-expression *Dnm1l* and *Mfn1*. (A)** Experimental design for Dnm1l and Mfn1 re-expression *in vivo*. AAV was injected to induce *Dnm1l* and *Mfn1* re-expression specifically in cardiomyocytes. **(B)** Echocardiographic measurement of myocardial thickness and contractile function. N = 6 hearts per group. **(C)** HE staining demonstrated significant reduced hypertrophy on DRP1 and MFN1 re-expressed AAC heart. **(D)** qPCR showed the significant increased expression of *Dnm1l* and *Mfn1* in cardiac tissue. N = 3 hearts per group. **(E)** Cross-section with WGA staining of AAC and treated hearts. Box plots demonstrated the individual and combine infection rate of two AAV vectors. **(F)** *In situ* optical sections and quantification between AAC and treated hearts indicating reduced cell size on bi-infected cardiomyocytes, revealing a cell autonomous manner on cell size by re-expression of *Dnm1l* and *Mfn1*. **(G)** MitoTracker staining and quantification of mitochondria morphology in YAP1-overexpressing NMVMs upon MFN1 and DRP1 addback by AdVs. N = 3 biological replicates in each group. **(H)** MitoSox density and quantification of mitochondrial ROS level in YAP1-overexpressing NMVMs upon MFN1 and DRP1 addback by AdVs. N = 3 biological replicates in each group. **(I)** OCR measurement of NMVMs normalized to cell number. N = 9 hearts per group. Mean ± SD. scale bars, 50 µm. Student's t-test or Mann-Whitney U-test was applied. *P < 0.05. **P < 0.01. ***P < 0.001. ****P < 0.0001. Non-significant P values in parenthesis.

Next, we used AAV and AdV to increase the expression of DRP1 and MFN1 in AAC mice (*in vivo*) and YAP1-overexpressing NMVMs (*in vitro*). In the *in vivo* model, AAV was injected into mice at P28 at high-dose of 4×10^10^ vg/g. The co-injection of AAV-cTNT-GFP and AAV-cTNT-mScarlet were used as controls, while the co-injection of AAV-cTNT-Dnm1l-GFP and AAV-cTNT-Mfn1-mScarlet were used for treatment. AAC was then induced at P30 and analyses were performed at P90 (Figure 6A). We performed echocardiographic and histological analyses and observed a decrease in ventricular wall thickness after treatment (Figure 6B-C). At the same time, the LVEF and FS remained unchanged (Figure 6B). H&E staining also demonstrated the inhibition of hypertrophy by the re-expression of AAV-derived DRP1 and MFN1 in AAC mice (Figure 6C). The upregulation of *Dnm1l* and *Mfn1* was confirmed by qPCR (Figure 6D) and immunoblotting (Figure S12A). The cross-section with WGA staining of AAC alone and AAV-treated hearts demonstrated the co-infection ratio of AAV-cTNT-Dnm1l-GFP and AAV-cTNT-Mfn1-mScarlet (Figure 6E). Generally, the individual infection ratio of *Dnm1l* or *Mfn1* was around 65% in both AAC alone and AAV-treated mice. While the co-infection ratio of double positive on GFP and mScarlet cardiomyocytes ranged from 40% to 60%, indicating that ~50% of cardiomyocytes were co-infected with AAV-cTNT-Dnm1l-GFP and AAV-cTNT-Mfn1-mScarlet. Notably, cardiomyocytes hypertrophy was attenuated after the re-expression of *Dnm1l* and *Mfn1 in vivo*, which was validated by the reduced cardiomyocyte cross-sectional area in the co-infected cardiomyocytes of AAC myocardial sections (Figure 6E and Figure S12B) and *in situ* optical sections analyzed by confocal microscopy (Figure 6F). Moreover, we found that only the re-expression *Dnm1l* and *Mfn1* together could prevent the induction of cardiac hypertrophy by chronic mechanical overloading in a cell autonomous manner. We used qPCR to validate the overexpression of *Dnm1l* and *Mfn1* by AdVs *in vitro* (Figure S12C). Here, YAP1-induced cardiomyocyte hypertrophy was also significantly attenuated by the AdV-mediated add-back of DRP1 and MFN1 (Figure S12D). We also found that the mitochondrial morphological disorganization was partially rescued (Figure 6G) and the MitoSox density was reduced after the re-expression of *Dnm1l* and *Mfn1* (Figure 6H). Importantly, co-expressing DRP1 and MFN1 was sufficient to elevate the OCR of YAP1-overexpressing NMVMs to nearly the control level (Figure 6I). Together, such *in vivo* and *in vitro* data show that the combined loss of DRP1 and MFN1 was responsible for the YAP1-induced cardiomyocyte hypertrophy.

### Verteporfin can attenuate AAC-induced pathological hypertrophy

**Figure 7:**
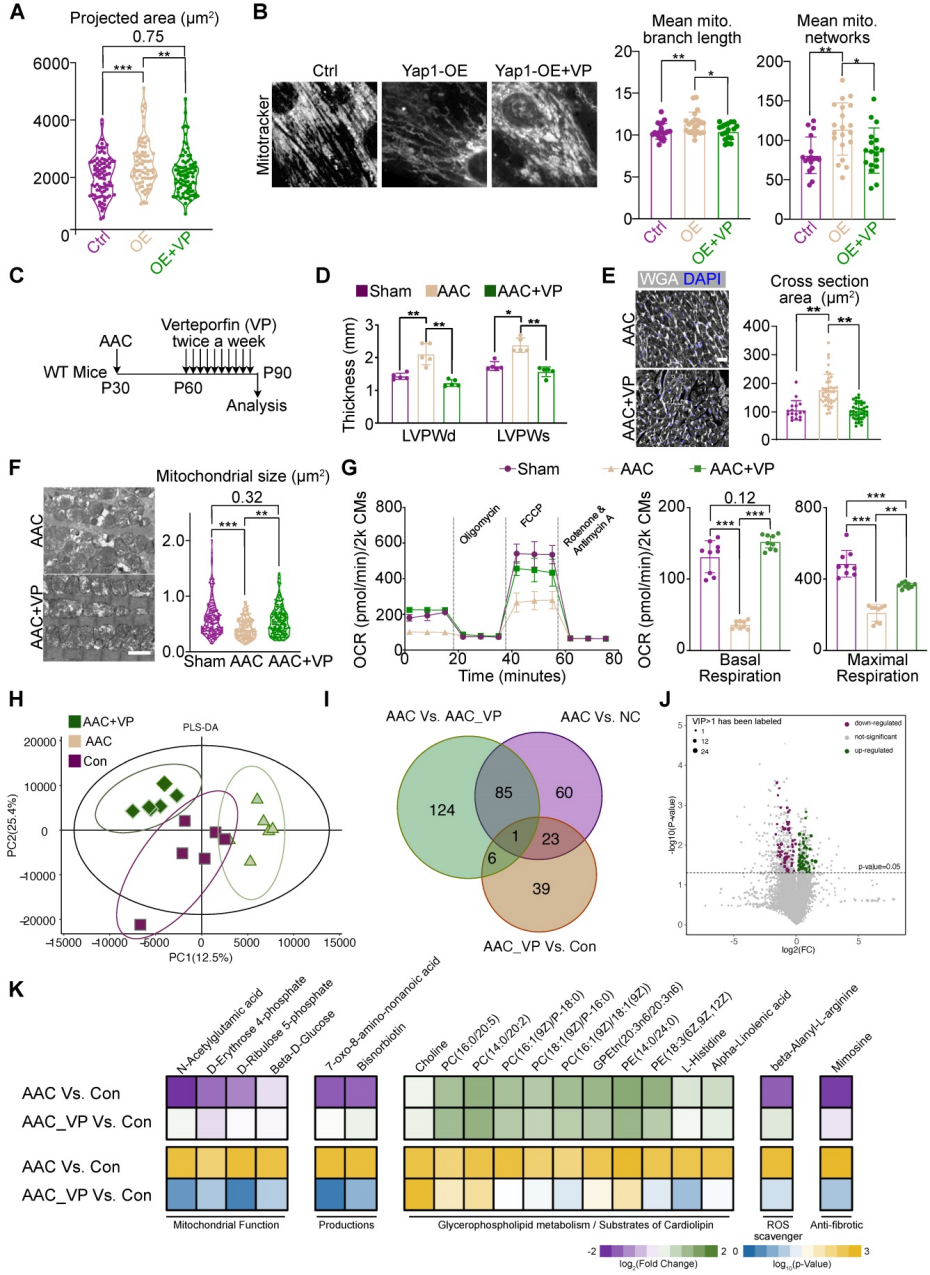
** Verteporfin attenuates AAC-induced pathological hypertrophy. (A)** Projected cell area of YAP1-overexpressing NMVMs after verteporfin treatment. N = 3 biological replicates in each group.** (B)** MitoTracker staining and quantification of mitochondria morphology in NMVMs after verteporfin treatment. Scale bar, 50 µm. N = 3 biological replicates in each group. **(C)** Experimental design to test the impact of verteporfin on AAC-induced pathological hypertrophy in mice. **(D)** Echocardiographic measurement of LVPW thickness in control, AAC-treated and verteporfin-treated mice. N = 5 mice per group. **(E)** WGA staining and cardiomyocyte cross section area measurement in heart sections. Scale bar, 50 µm. N = 3 hearts per group. **(F)** TEM imaging and measurement of mitochondria cross section area. Scale bar, 2 µm. N = 3 hearts per group. **(G)** OCR measurement of isolated cardiomyocytes normalized to cell number. Mean ± SD. N = 9 hearts per group. **(H)** PLS-DA distribution of metabolic substances profile after verteporfin administration based on LC-MS. N = 3 hearts per group. **(I)** Venn plot shows the overlaps of differentiated metabolic substances among three comparisons. N = 3 hearts per group. **(J)** Volcano plot demonstrates the differentiated metabolic substances with P < 0.05 and VIP > 1. N = 3 hearts per group. **(K)** Heatmap of representative metabolic substances between the two comparisons. Upper panel shows the log2 of fold change and lower panel shows the log10 of P value. N = 3 hearts per group. Student's t-test applied or Mann-Whitney U-test was applied. *P < 0.05. **P < 0.01. ***P < 0.001. Non-significant P values in parenthesis.

Verteporfin is an FDA-approved drug that is reported to inhibit YAP signaling by multiple mechanisms 39, 40. We treated NMVMs overexpressing YAP1 with 10 μM verteporfin and found that verteporfin decreased YAP signaling after 48 hours of treatment (Figure S13A). Verteporfin also reduced the association between YAP1 and TEAD1, as supported by a co-immunoprecipitation experiment (Figure S13B). We examined the impact of verteporfin on YAP1-overexpressing NMVMs and observed that it attenuated cellular hypertrophy (Figure 7A) and rescued mitochondrial morphology (Figure 7B). The levels of DRP1, MFN1 and γH2AX in YAP1-overexpressing NMVMs were also partially rescued by verteporfin (Figure S13C-D).

We next administered verteporfin to mice undergoing AAC for a month and examined its impact on cardiac hypertrophy 2 months after the surgery (Figure 7C). We found that verteporfin significantly attenuated the hypertrophic growth of left ventricular walls (Figure 7D), while LVEF and FS remained unchanged upon verteporfin treatment (Figure S14A). The increase in cardiomyocyte cross-sectional area was also reversed (Figure 7E). Masson staining revealed a reduction in the fibrotic area after verteporfin administration to AAC mice (Figure S14B). Furthermore, the inhibition of fibrosis and the expression of cardiac stress markers such as *Ctgf*, *Nppa, Nppb* were also detected in verteporfin-treated ventricles (Figure S14C). TEM analysis showed that mitochondrial size was restored upon verteporfin administration in AAC-subjected myocardium (Figure S7F). The oxidative respiratory capacity of cardiomyocytes was also enhanced (Figure S7G). Thus, verteporfin could be potentially repurposed to treat pathological hypertrophy and hypertensive heart disease by targeting YAP signaling.

Next, RNA-seq and GSEA analysis demonstrated the inhibition of YAP signaling and ECM, as well as the restoration of mitochondrial function in verteporfin-treated mice compared to untreated AAC mice (Figure S14D). PCA and sample correlation analyses showed a tight clustering of features between sham and verteporfin-treated mice, which were significantly different from the transcriptional profile of untreated AAC hearts (Figure S14E-F). GO (Figure S15A) and KEGG (Figure S15B) enrichment analyses showed the upregulation of genes associated with cardiac maintenance and mitochondrial function, while those associated with the processes of fibrosis, inflammation, ECM remodeling were inhibited upon treatment of AAC mice with verteporfin.

To further investigate the changes in mitochondrial function and metabolic profile, LC-MS was used to identify critical metabolic substances detected upon verteporfin administration. Six heart sample replicates were evaluated consecutively for each group (AAC, AAC+VP, and Con groups). Varied metabolic profiles were found among the three groups based on PLS-DA analysis, indicating that verteporfin could regulate the metabolic status of cardiomyocytes (Figure 7H). There were 169 significantly changed substances between AAC and control myocardial tissues. However, only 69 substances could be retrieved from the AAC+VP and control tissues. The Venn diagram illustrates the overlapping of substances among the groups, and a few overlaps of substances were identified (Figure 7I). Volcano plots were used to reveal the downregulated and upregulated metabolic substances with P < 0.05 and VIP > 1 between AAC+VP and control groups (Figure 7J). A series of significantly changed substances were then presented according to their associated functions. The presence of mitochondrial function-related substances (including *N*-acetylglutamic acid, D-erythrose 4-phosphate, D-ribulose 5-phosphate, and beta-D-Glucose) was reduced in AAC mice but was recovered after verteporfin administration. Similar findings were observed for the metabolic substances category (e.g., 7-oxo-8-amino-nonanoic acid and bisnorbiotin). Reduction of the ROS scavenger, beta-alanyl-L-arginine, was identified in AAC mice. The anti-fibrotic substance mimosine was also re-expressed in the AAC+VP group (Figure 7K, left and right panels). However, there were almost no changes in the distribution of substances associated with glycerophospholipid metabolism or the substrate cardiolipin (Figure 7K, middle panel).

### Prolonged mitochondria dysfunction impairs YAP1-induced cardiomyocyte proliferation

**Figure 8:**
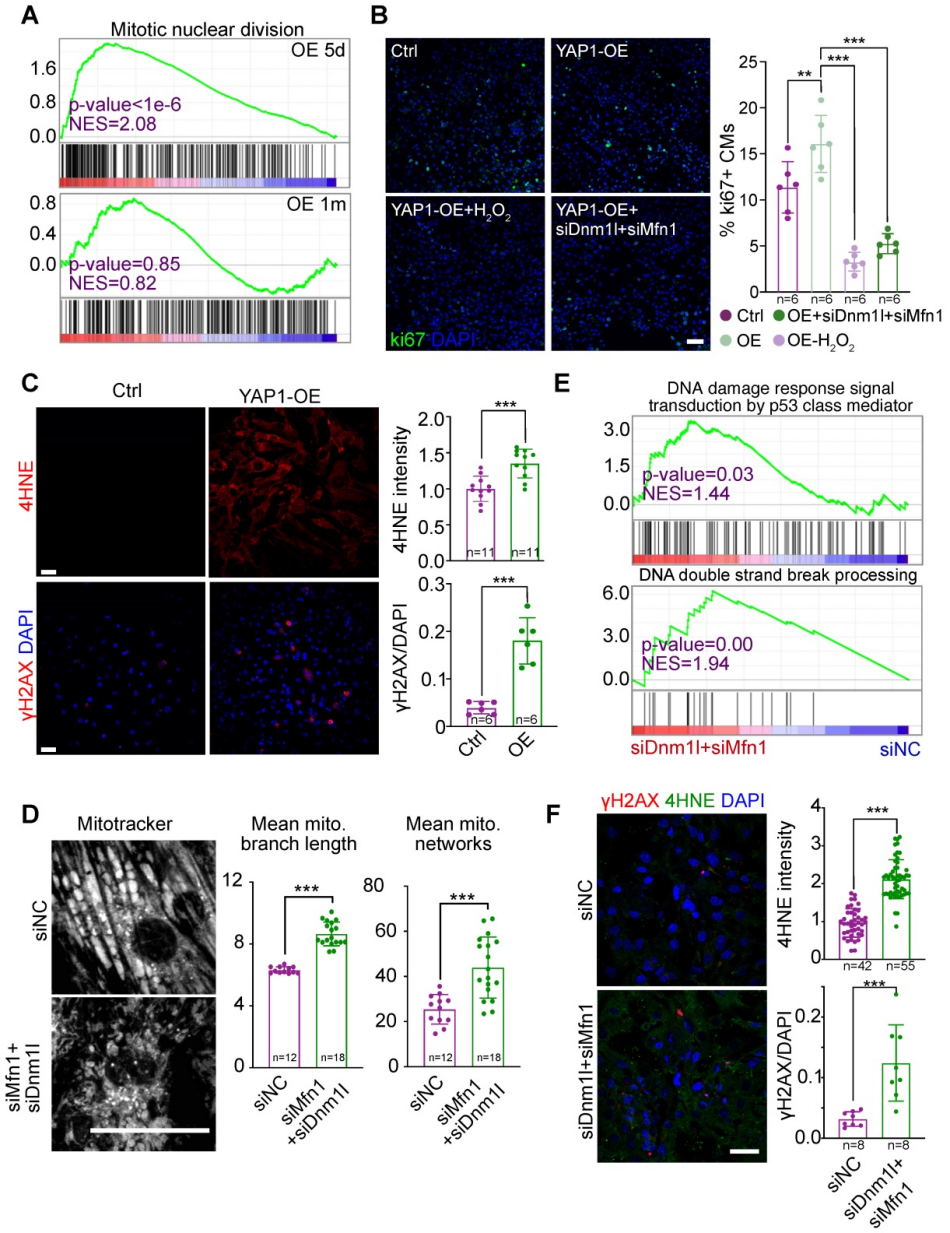
** Prolonged YAP1 overexpression suppresses cell cycle through mitochondrial damage-triggered ROS increase and DNA damages. (A)** GSEA of the mitotic division gene sets in RNA-Seq analysis for YAP1 overexpression in the heart. **(B)** Ki67 staining on NMVMs overexpressing YAP1 and quantification. N indicates the number of biological repeats. **(C)** 4HNE and γH2AX staining on NMVMs overexpressing YAP1 and quantification.** (D)** MitoTracker staining and quantification of mitochondria morphology in NMVMs upon *Dnm1l* and *Mfn1* RNAi silencing. N = biological replicates in each group. **(E)** GSEA of DNA damage gene sets in RNA-Seq analysis for *Dnm1l-Mfn1* knockdown in the heart. **(F)** 4HNE and γH2AX staining and quantification on NMVMs upon Dnm1l and Mfn1 RNAi silencing. N indicates the number of biological repeats or cells. Mean ± SD. Student's t-test applied or Mann-Whitney U-test was applied. **P < 0.01. ***P < 0.001. In fluorescence images, scale bars stand for 50 µm. N indicates the number of biological repeats.

YAP1 is a well-established stimulator of the cell cycle. However, in our RNA-seq analysis, cell division-related gene sets were only up-regulated in the hearts of mice overexpressing YAP1 for 5 days, but not for 1 month (Figure 8A). We previously reported that mitochondrial dysfunction could increase ROS, cause DNA damage, and thereby inhibit the cardiomyocyte cell cycle 19. To test if this pathway contributed to cell cycle inhibition in YAP1-overexpressing cardiomyocytes, we induced NMVM proliferation by overexpressing YAP1 and then knocked down *Dnm1l* and *Mfn1* by RNAi*.* By analyzing the cell proliferation marker Ki67, we found that *Dnm1l* and *Mfn1* knockdown was sufficient to inhibit the YAP1-induced cell cycling in NMVMs after 3 days of culture (Figure 8B). YAP1 overexpression induced the expression of the ROS marker 4HNE and the DNA damage marker γH2AX in NMVMs by day 7 of culture (Figure 8C). MitoTracker staining was used to demonstrate the impairment of mitochondrial morphology by *Dnm1l-Mfn1* knockdown (Figure 8D). Moreover, GSEA demonstrated that DNA damage occurred after this *Dnm1l*-*Mfn1* knockdown (Figure 8E). Besides, *Dnm1l*-*Mfn1* knockdown induced 4HNE and γH2AX expression in NMVMs by day 3 of culture (Figure 8F). These data show that the mitochondrial dysfunction induced by *Dnm1l-Mfn1* inhibition could silence the cell division program that was initially induced by YAP1 overexpression.

## Discussion

Cardiac hypertrophy is a major pathological feature of heart disease. Understanding the mechanisms that regulate cardiac hypertrophy is crucial to uncover gene targets for cardiac therapy. In this study, we performed AAC surgery in mice to induce chronic pressure overload and pathological hypertrophy in the heart. We found that YAP activation was a major driver of AAC-induced pathological hypertrophy. This novel role of YAP1 was shown to be mediated by mitochondria damage, depending on the duration of YAP1 activation. Interestingly, verteporfin, an FDA-approved inhibitor of YAP signaling, was found to attenuate AAC-induced hypertrophy. Thus, this study not only provided proof-of-concept evidence that YAP1 was an effective therapeutic target, but also pointed out a compound that could be repurposed to treat hypertensive heart diseases.

Pressure overload-induced cardiac hypertrophy was known to be associated with the remodeling of mitochondria and the decrease in oxidative respiration. However, the links between pressure, hypertrophy, and mitochondria were poorly understood. YAP1 is a well-established mediator of mechanical stress-induced signals 41, 42, which responds to pressure overload in the heart. In recent years, several mechanotransduction pathways have been identified as being involved in the regulation of cardiomyocytes, and a series of links have been found between mechanical stress and mitochondrial function. Mechanical stress was demonstrated to lead to the impairment of mitochondrial quality control and the electron transport chain, induce ROS accumulation, and result in Ca^2+^ mis-regulation 43. In addition, research from our group also identified a novel relationship between mitochondrial function and YAP1 activation 44. Moreover, all the adverse changes affecting mitochondria contribute to cardiac hypertrophy. Our earlier study demonstrated that AAV administration in a non-cell-autonomous manner could be used to attenuate cardiomyocyte hypertrophy by targeting paracrine signaling via the ECM to reduce viral dosage. We found that the reduction in mechanical stress as a result of ECM stiffening participated in the regulation cardiomyocyte hypertrophy 45. However, how the mechanotransduction signal regulates mitochondria and its impact on cardiac hypertrophy had yet to be elucidated.

In this study, we showed that prolonged YAP1 activation was sufficient to trigger hypertrophy through the perturbation of mitochondria. Strikingly, the simultaneous restoration of a mitochondrial fission activator DRP1 and a mitochondrial fusion activator MFN1 was necessary to rescue YAP-induced mitochondrial defects and cardiac hypertrophy, but overexpression of DRP1 or MFN1 alone did not. These data show that a balance between mitochondrial fission and fusion is required for cardiac health. Restoration of this balance is required to rebuild the respiratory health in a heart experiencing pathological hypertrophy. This present study has therefore linked the mechanotransduction molecule, YAP1, with mitochondrial function. A series of RNA-seq analyses were used to identify mitochondrial dysfunction as the major adverse effect of overload pressure, showing that mitochondrial biogenesis was impaired, leading to hyperfragmention. ATAC-seq and TEAD1 bioChIP-seq were performed to determine how the role of the TEAD1-YAP1 complex, and revealed that this complex regulated the expression of DRP1 and MFN1. Phenotypic rescue was achieved by the re-expression of DRP1 and MFN1 following YAP1 activation. These data suggest that YAP1 activation causes mitochondrial disorder in a manner dependent on TEAD1-mediated transcription regulation. Recent research from Liu et al. confirmed that TEAD1 is essential for mitochondrial function, which is similar to the results of the luciferase assays generated in the present study. Furthermore, Leach et al. reported that Hippo pathway deficiency could reverse heart function by regulating mitochondrial homeostasis via *Park2* 46. Accordingly, YAP1 was proven to be involved in mitochondrial function; however, this is the first study to demonstrate a clear regulatory link between YAP1, mitochondrial biogenesis, and combined *Dnm1l* and *Mfn1* gene expression.

Verteporfin was developed as a type of small-molecule inhibitor of YAP1. Numerous studies have confirmed the inhibition of YAP1 by verteporfin in various processes ranging from cancer to wound scarring 47. The most recent research by Mascharak et al. has demonstrated the therapeutic effects of verteporfin in enabling wound regeneration without scarring by inhibiting YAP1 in fibroblasts 48. Our data confirm that the administration of verteporfin can inhibit YAP signaling and restore mitochondrial function. Collectively, verteporfin elicits promising pharmaceutical effects by terminating excessive mechanical stress.

YAP signaling is widely studied as a target for promoting cardiac regeneration and repair. However, recent studies have started to uncover the novel roles of YAP signaling in promoting cardiac pathogenesis and damage, raising concerns about YAP-targeted cardiac therapy 15, 49. In our study, we confirmed the role of YAP1 activation in promoting cardiomyocyte cell cycling in adult cardiomyocytes after 5 days of YAP1 overexpression. However, prolonged YAP1 activation overrode this effect and caused mitochondria to elicit ROS-mediated DNA damage. Thus, our work reconciled the seemingly conflictive findings that YAP both promotes cardiac repair and cardiac damage, and highlighted that the temporal control of YAP1 activation is a key factor that determines the success in YAP1-targeted cardiac therapy.

In summary, our data demonstrate that mechanical stress overload induces YAP activation and causes mitochondrial biogenesis impairment via the inhibition of *Dnm1l* and *Mfn1* co-expression. Therefore, the inhibition of *Yap1* helps maintain the normal morphology of cardiomyocytes, meaning that verteporfin represents a promising candidate for treating cardiac hypertrophy.

## Supplementary Material

Supplementary figures.
