# Supplementary material for: Yap1 modulates cardiomyocyte hypertrophy via impaired mitochondrial biogenesis in response to chronic mechanical stress overload
Source: Theranostics. 2022 Oct 3;12(16):7009–31. doi: 10.7150/thno.74563 (PMC9576622; doi:10.7150/thno.74563)
Supplement: Supplementary file 1 — Supplementary figures. [file thnov12p7009s1.pdf]

Supplementary Materials for

Yap1 modulates cardiomyocyte hypertrophy via impaired mitochondrial biogenesis in response to chronic mechanical stress overload

Running title: YAP1 promotes cardiac hypertrophy

Peng Yue[1]#, Yue Zhang[1]#, Lei Liu[1]#, Kaiyu Zhou[1]#, Shutao Xia[2], Mou Peng[1], Hualin Yan[3], Xiaoqiang Tang[1], Zhan Chen[4], Donghui Zhang[2], Junling Guo[5], William T. Pu[6,7], Yuxuan Guo[4]†, Yimin Hua[1]†, Yifei Li[1]†

1. Key Laboratory of Birth Defects and Related Diseases of Women and Children of MOE, Department of Pediatrics, West China Second University Hospital, Sichuan University, Chengdu, Sichuan 610041, China.
2. State Key Laboratory of Biocatalysis and Enzyme Engineering, School of Life Science, Hubei University, Wuhan, Hubei 430062, China.
3. Department of Medical Ultrasound, West China Hospital, Sichuan University, Chengdu, Sichuan 610041, China.
4. Peking University Health Science Center, School of Basic Medical Sciences, The Institute of Cardiovascular Sciences, Key Laboratory of Molecular Cardiovascular Science of Ministry of Education, Beijing Key Laboratory of Cardiovascular Receptors Research, Beijing, 100191, China.
5. BMI Center for Biomass Materials and Nanointerfaces, College of Biomass Science and Engineering, Sichuan University, Chengdu, Sichuan 610065, China.
6. Department of Cardiology, Boston Children’s Hospital, Boston, MA 02115 USA.
7. Harvard Stem Cell Institute, Harvard University, Cambridge, MA 02138 USA.

* These authors contributed equally to this work.

†Correspondence to:
Yuxuan Guo, PhD, Peking University Health Science Center, School of Basic Medical Sciences, The Institute of Cardiovascular Sciences, Key Laboratory of Molecular Cardiovascular Science of Ministry of Education,38 Xueyuan Road, Beijing, 100191, China. Email: guo@bjmu.edu.cn;

Yimin Hua, MD, Key Laboratory of Birth Defects and Related Diseases of Women and Children of MOE, Department of Pediatrics, West China Second University Hospital, Sichuan University, 20 3rd Section, Renmin S.Rd., Chengdu, Sichuan, 610041. Email: nathan_hua@163.com;

Yifei Li, MD, Key Laboratory of Birth Defects and Related Diseases of Women and Children of MOE, Department of Pediatrics, West China Second University Hospital, Sichuan University, 20 3rd Section, Renmin S.Rd., Chengdu, Sichuan, 610041. Tel: 86-181-8060-1016. Email: liyfwcsh@scu.edu.cn. (Leading contact)

This PDF file includes:

Figure S1 to S15

Figure S1

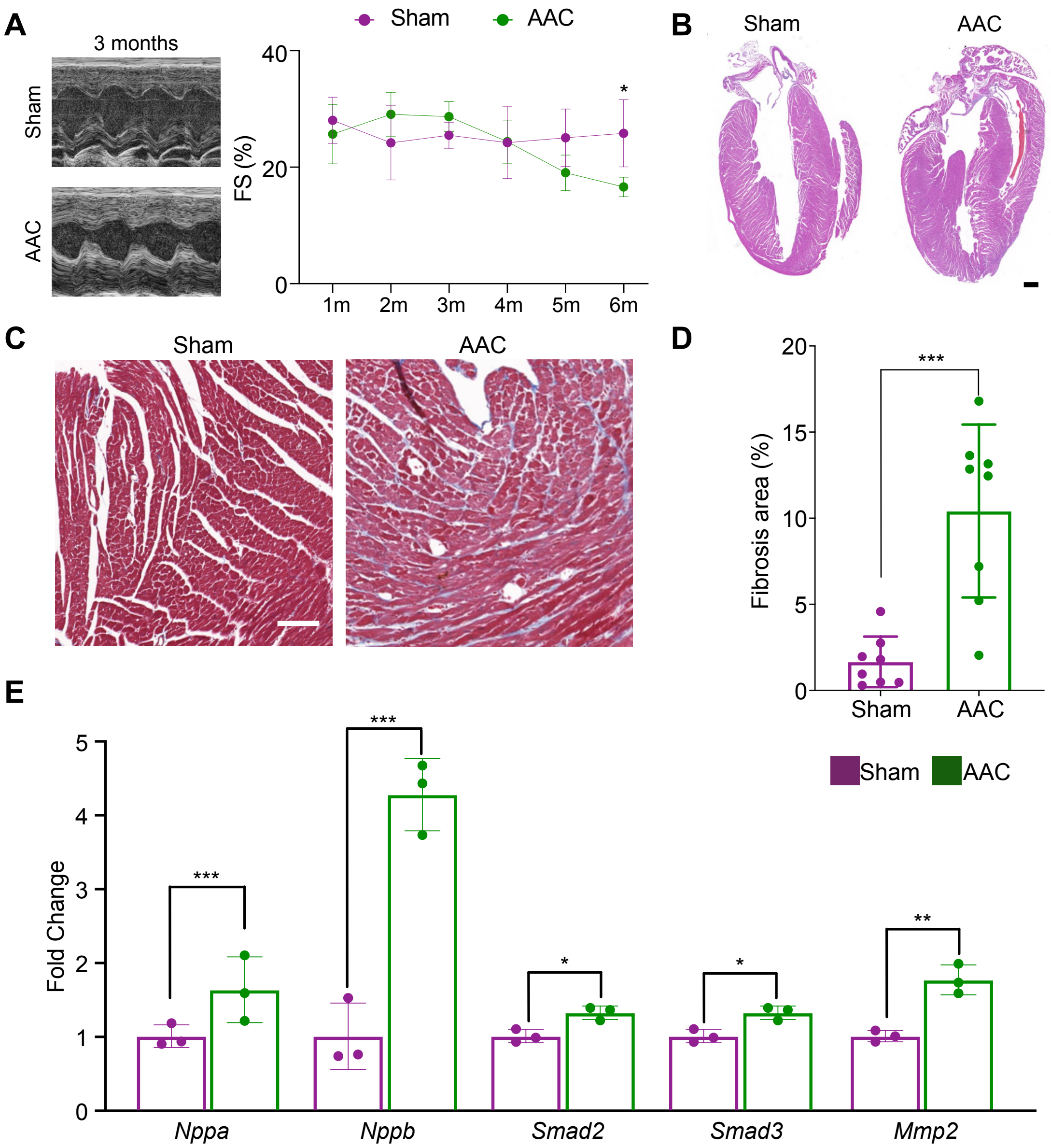

Figure S1. AAC induces cardiac hypertrophy and interstitial fibrosis in the heart. (A) The echocardiographic analysis of fraction shortening (FS) in sham- or AAC-treated mice with presentative M-model images by two months post surgery. Mean ± SD. N = 12. **(B)** HE staining on cardiac longitudinal section at the end of 2nd month after AAC. **(C-D)** Masson staining on cardiac sections at the 2nd month after AAC surgeries and quantification of the relative fibrotic area. N = 8 hearts. Scale bar, 200 μm. **(E)** RT-qPCR analysis of cardiac remodeling and fibrotic genes in hearts subjected to AAC. N = 3 hearts. Scale bar, 50 μm. In statistical analysis, student's t-test was applied.*P < 0.05, **P < 0.01, ***P < 0.001.

Figure S2

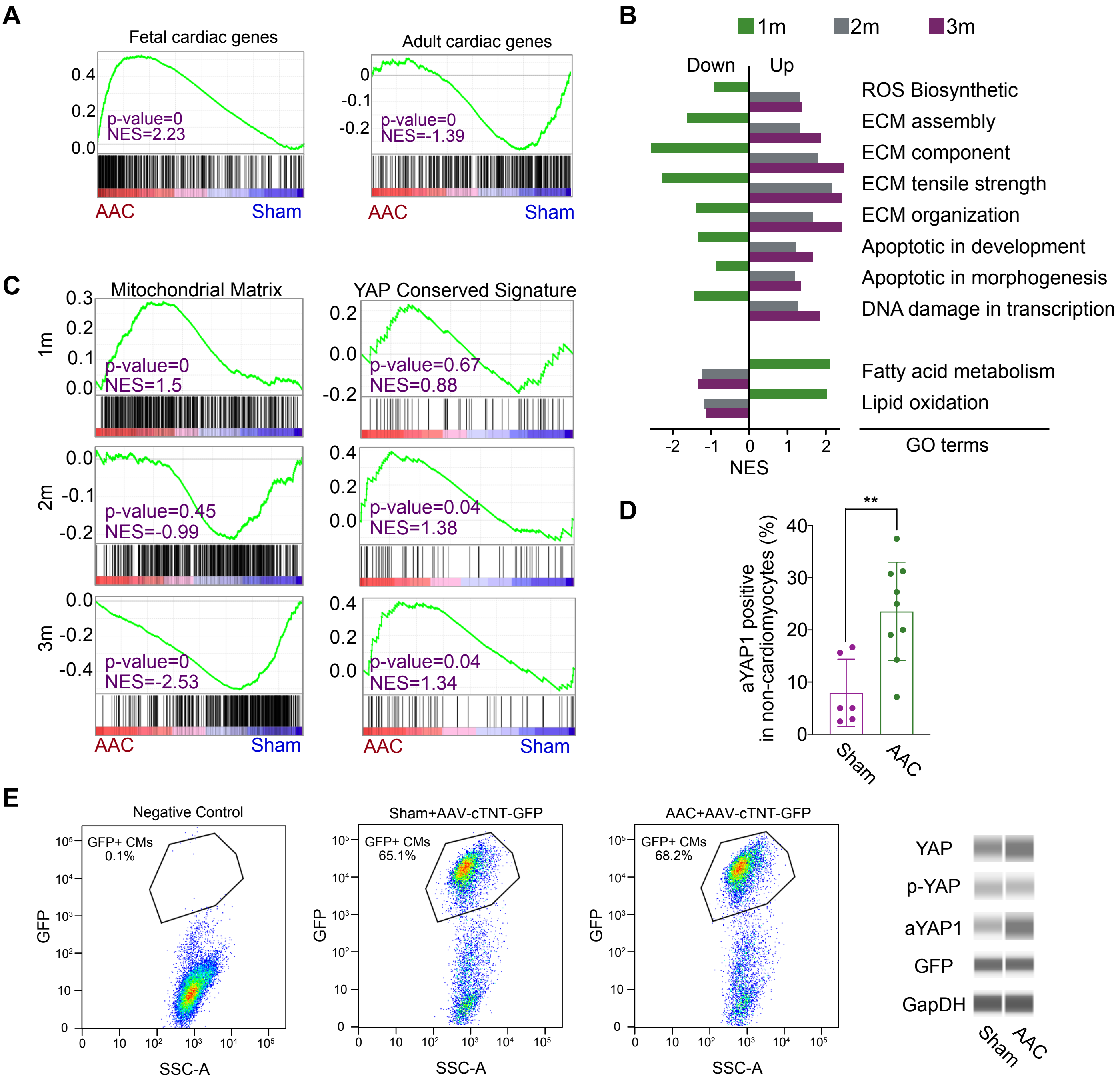

Figure S2. AAC induces the downregulation of mitochondrial genes associated with YAP1 activation. (A) GSEA analysis of RNA-seq data for hearts treated by AAC for 2 months. **(B-C)** Comparative GSEA analysis of RNA-seq data for hearts treated by AAC for 1, 2 and 3 months. NES, normalized enrichment score. **(D)** Immunofluorescence staining for activated YAP (aYAP) of and quantification of aYAP+ non-cardiomyocytes in cardiac tissue sections. **(E)** FACS sorting results for GFP+ cardiomyocytes by the two month after AAV applied to AAC and sham mice.Then the sorted cardiomyocytes were used to perform immunoblotting by WES. Mean ± SD. In statistical analysis, student's t-test was applied. **P < 0.01.

Figure S3

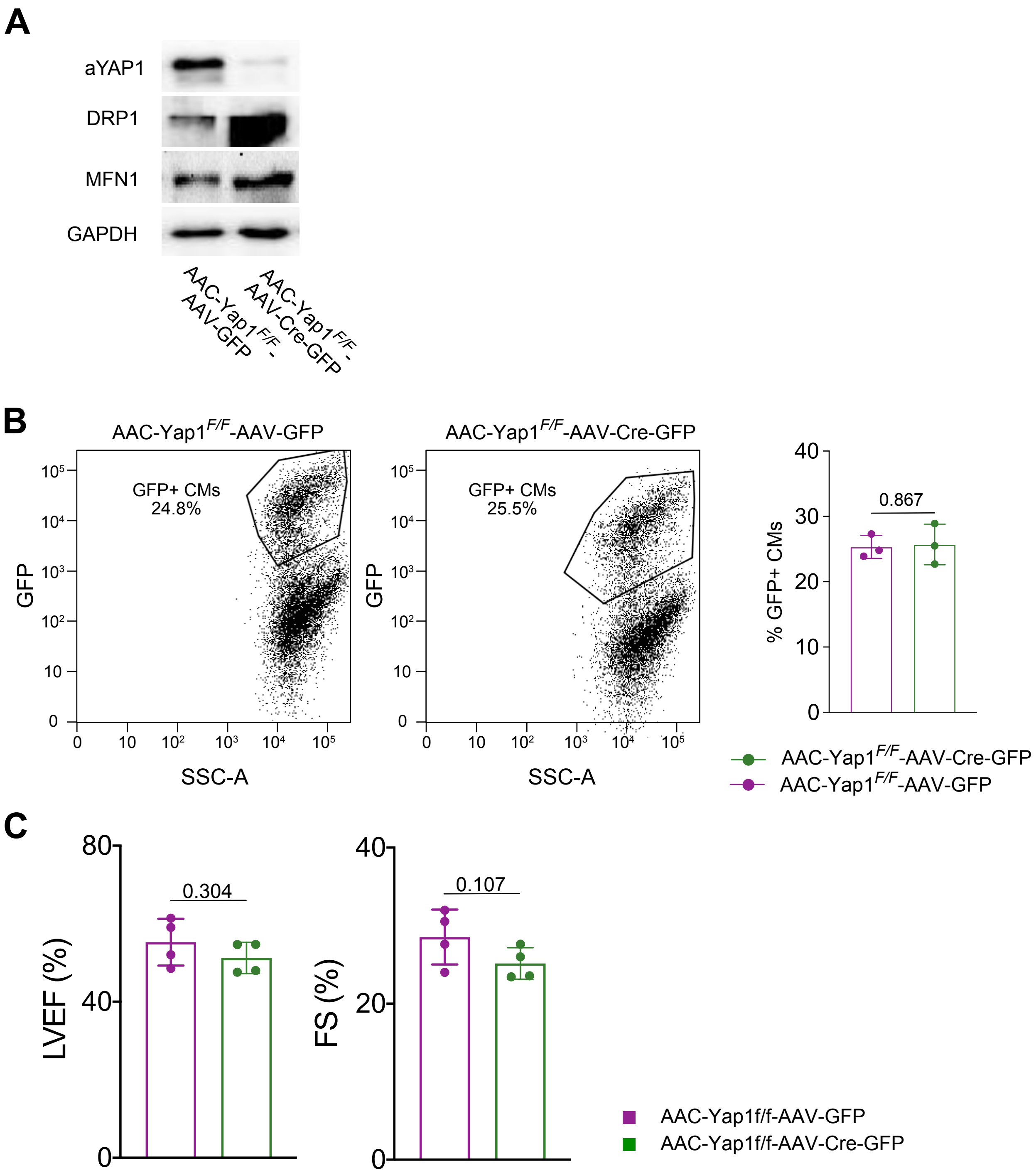

Figure S3. Depletion of YAP1 helps to maintain cardiac morphology. (A) Immunoblotting for activated YAP1 (aYAP1), DRP1 and MFN1 in cardiac tissue. **(B)** FACS sorting results for GFP+ cardiomyocytes by the one month after AAV applied to AAC mice. **(C)** Echocardiographic measurement of LVEF and FS in AAC-treated *Yap1*$^{F/F}$ mice. Mean ± SD. N = 4 hearts per group. In statistical analysis, student's t-test was applied. Non-significant P values in parenthesis.

Figure S4

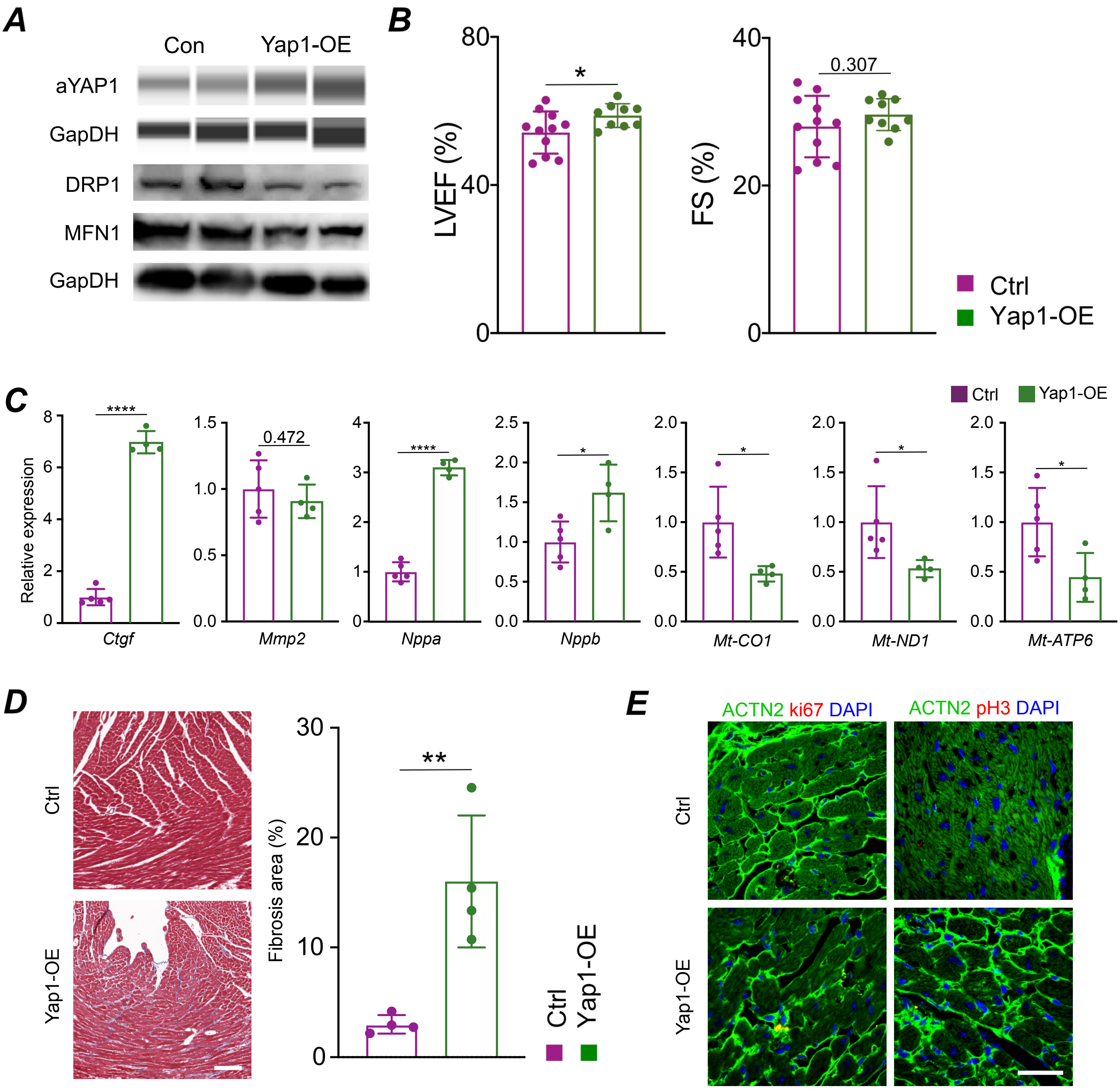

Figure S4. YAP1 activation induced HFpEF and failed to trigger proliferation in 1 month. (A) Immunoblotting demonstrated the YAP1 activation in vivo and DRP1 and MFN1 were inhibited. Tamoxifen was injected intraperitoneally to induce *Yap1* overexpression specifically in cardiomyocytes. **(B)** The echocardiographic analysis of LVEF and FS in Yap1-overexpression and control mice. Mean ± SD. N = 9. **(C)** RT-qPCR analysis of cardiac remodeling and fibrotic genes in hearts by YAP1-overexpression. N = 3 hearts. **(D)** Masson staining on cardiac sections at the one month after YAP activation and quantification of the relative fibrotic area. N = 4 hearts. Scale bar, 200 μm. **(E)** Immunostaining for ki67 and pH3 in cardiac tissue sections. Scale bar, 50 μm. In statistical analysis, student's t-test was applied.*P < 0.05, **P < 0.01, ****P < 0.0001. Non-significant P values in parenthesis.

Figure S5

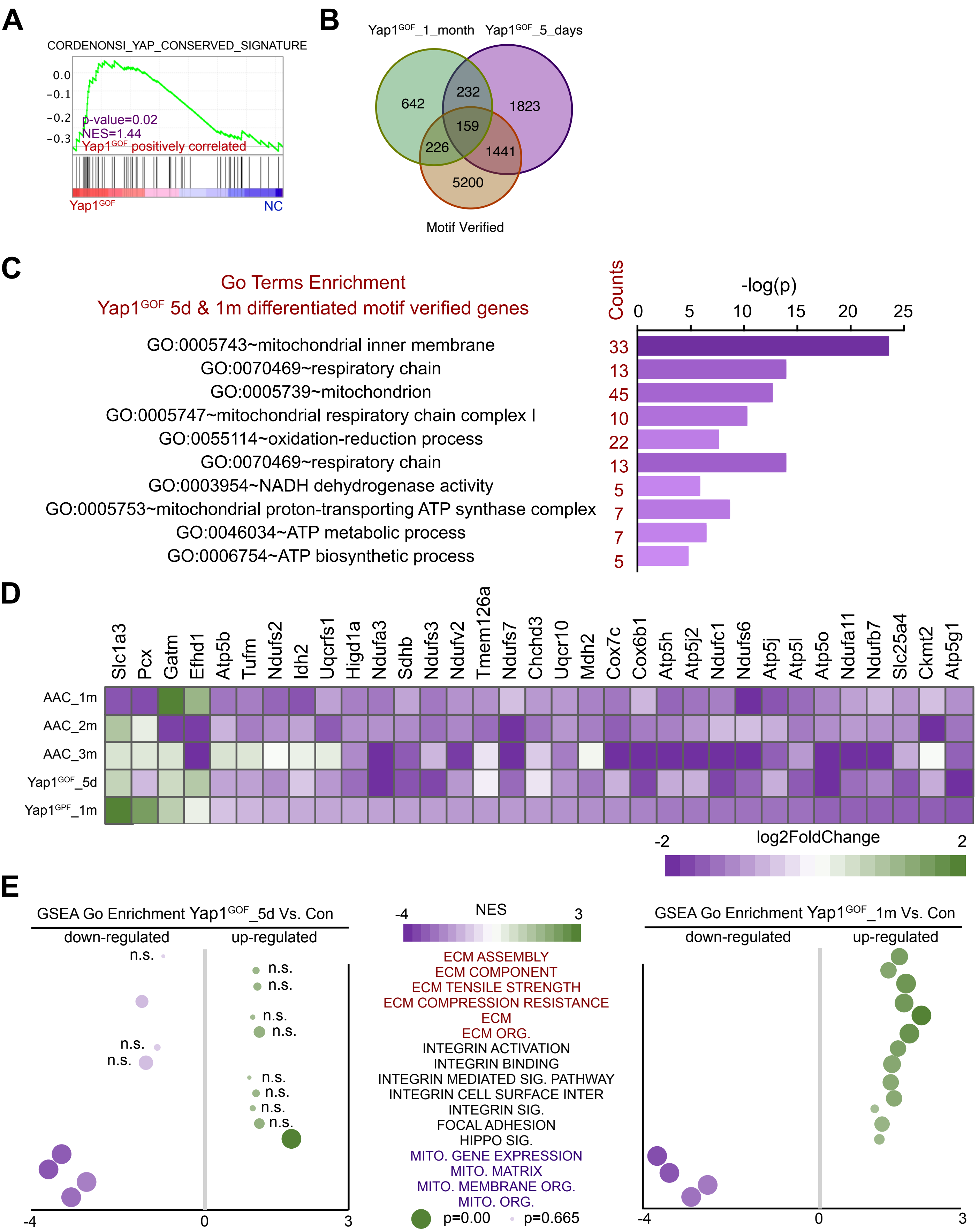

Figure S5. Transcription changes after cardiomyocyte specific YAP1 activation. (A) GSEA demonstrated the activation of YAP1 conserved signature by one month. **(B)** Venn diagram shows the overlap of differential expressed genes on 5 days and 1 month post YAP1 activation with the TEAD1 motif identified genes according to ATAC-seq. **(C)** The diagram shows the top 10 enriched GO terms of differentiated expressed genes of Yap1^{GOF} cardiomyocytes both identified of short term (5 days) and long term (1 month) with TEAD1 motif identification. **(D)** The heatmap of mitochondrial inner membrane related genes based on differentiated expressed genes of RNA-seq, indicating similar profile among AAC and YAP1 activation. **(E)** GO terms enriched among differentiated expressed genes between 5 days and 1 month upon YAP activation of Yap1$^{Myh6\text{-}GOF}$ hearts. N = 3 per groups.

Figure S6

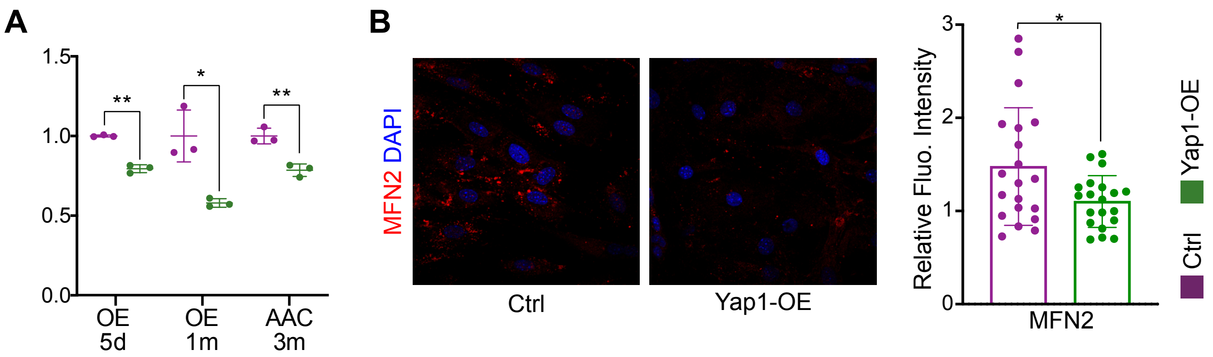

Figure S6. Mfn2 reduced after YAP1 activation. Mfn2 were measured by quantitative PCR **(A)** and immunostaining **(B)**. *Mfn2* had markedly decreased expression upon YAP activation both in heart tissues and cultured NMVMs**.** Scale bar, 50 μm. N = 3 biological replicates in each group. Mean ± SD. Student's *t*-test was applied. *P < 0.05. **P < 0.01. Non-significant P values in parenthesis.

Figure S8

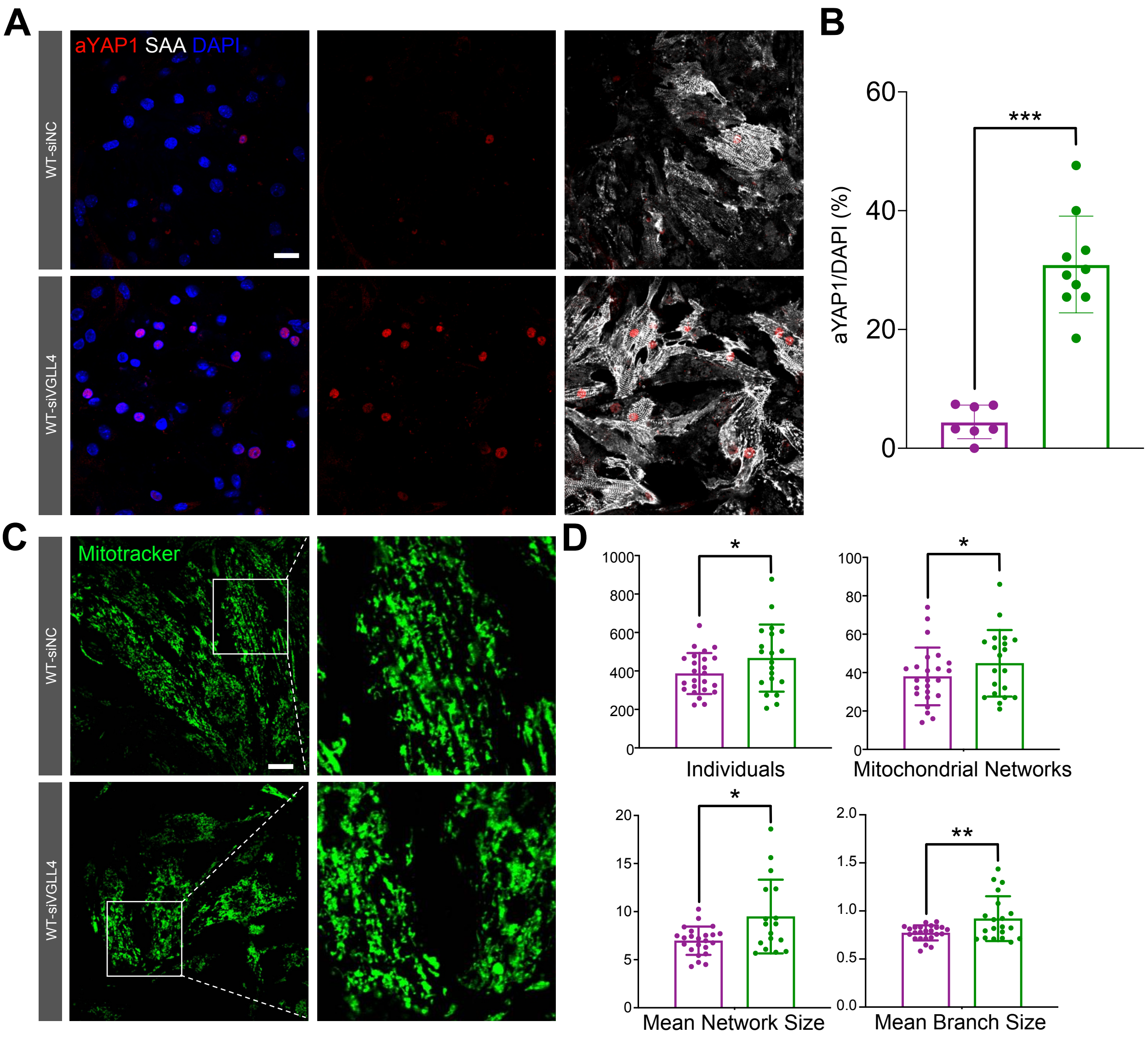

Figure S8. Vgll4 inhibition activates YAP1 and causes mitochondrial hyperfragmentation. (A) Elevated a-YAP+ cardiomyocytes after inhibition of Vgll4. **(B)** Quantitative analysis of a-YAP+ cardiomyocytes between control and siVGLL4 cardiomyocytes (N = 5 biologically independent samples and positive ratio of aYAP1 measured in 10 fields/slice). **(C)** MitoTracker staining of cardiomyocytes after *Vgll4* siRNA knock-down and control CMs. **(D)** Quantitative assessments of mitochondrial morphology, presenting a hyper-fragment change post *Vgll4* inhibition. Bar 50 μm; Student's t-test applied. *P < 0.05. **P < 0.01. ***P < 0.001.

Figure S9

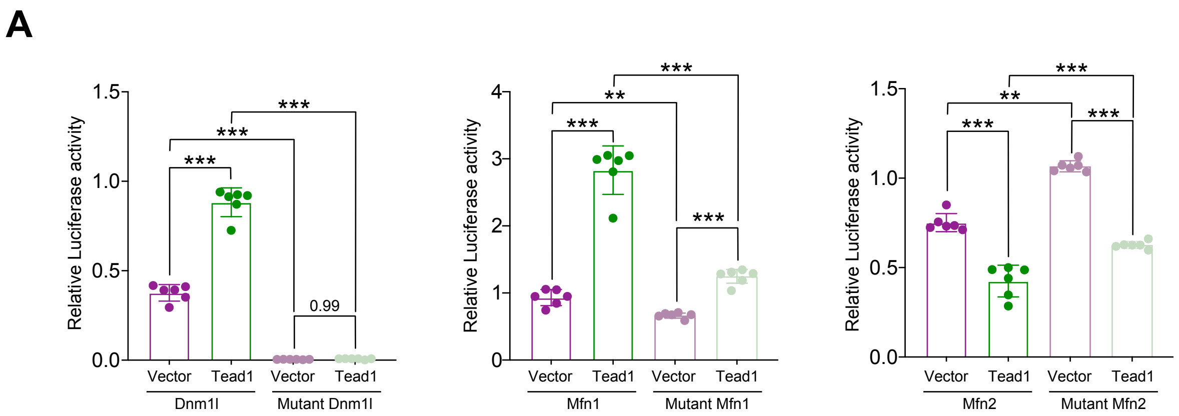

Figure S9. *Dnm1l* and *Mfn1* were regulated by TEAD1 complex. (A) The putative binding sites located in the promoter of *Dnm1l* and *Mfn1* and the experimental design of luciferase reporter. 293T cells were co-transfected of *Dnm1l/Mfn1*-WT or *Dnm1l/Mfn1*-MUT and Tead1 or control vectors. The luciferase activity was analyzed. N = 6 biological replicates in each group. Student's t-test applied. *P < 0.05. **P < 0.01. ***P < 0.001.

Figure S10

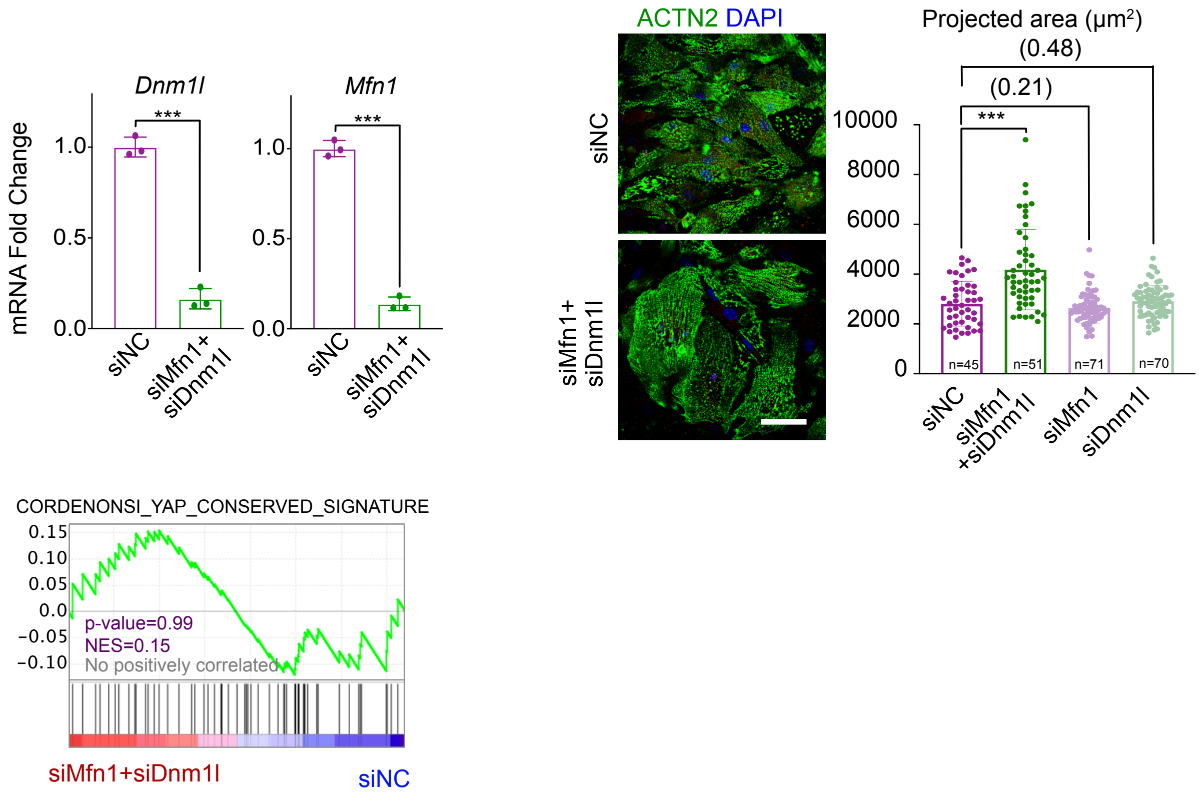

Figure 10. *Dnm1l* and *Mfn1* double inhibition causes cardiomyocytes hypertrophy. (A) RNAi has been used to inhibit the expression of *Dnm1l* and *Mfn1* at the same time in NMVMs. qPCR analysis the mRNA fold change after siRNA infected. **(B)** Leading edge analysis based on GSEA of RNA-seq on *Dnm1l* and *Mfn1* inhibited CMs, indicating the absence of enrichments of YAP signaling**.** (**C)** ACTN2 staining and projected cell area in NMVMs upon Dnm1l and Mfn1 RNAi silencing. N = 3 biological replicates in each group. Scale bar, 50 μm. In statistical analysis, student's t-test was applied. ****P < 0.0001. Non-significant P values in parenthesis.

Figure S11

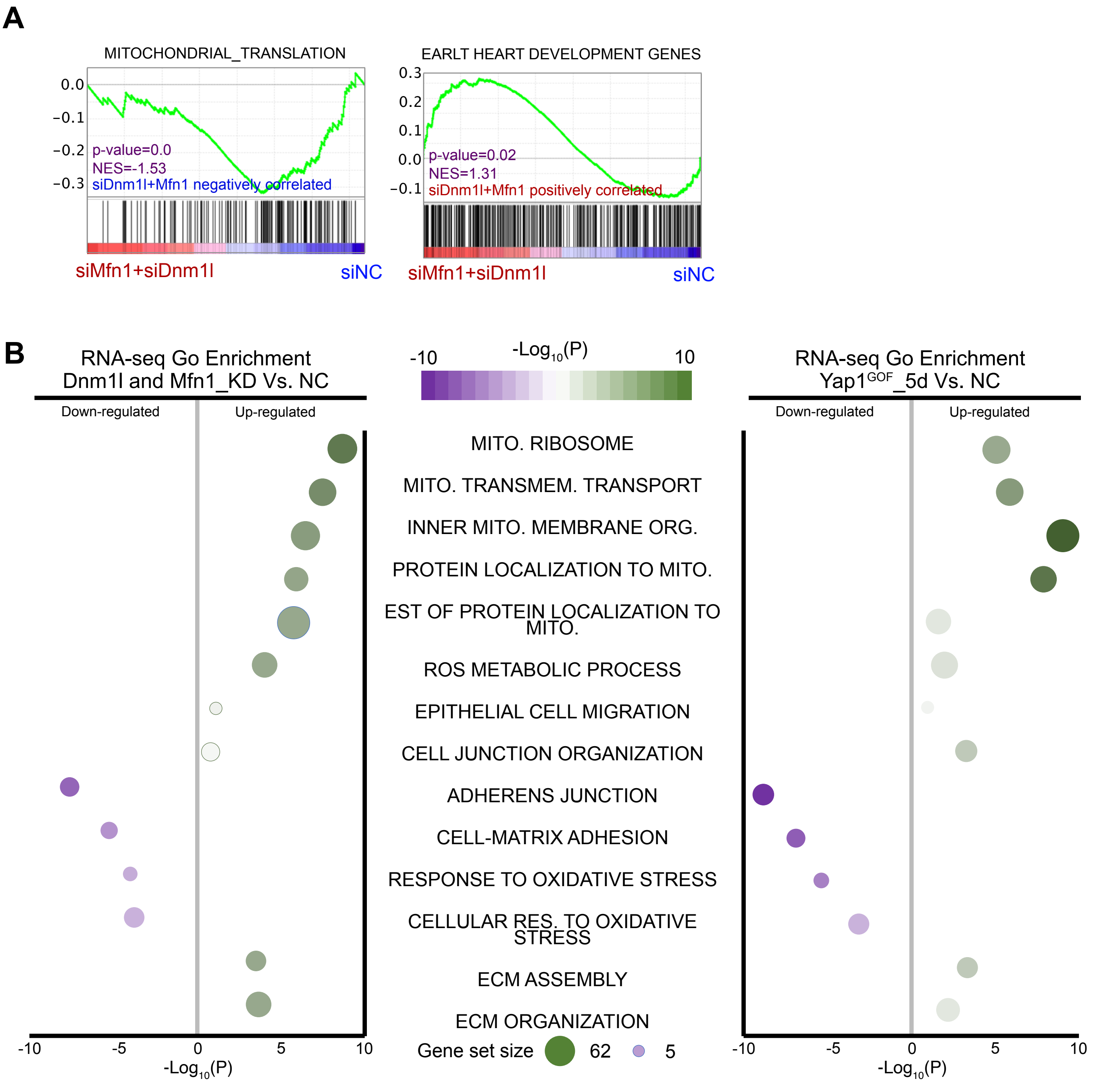

Figure S11. *Dnm1l* and *Mfn1* double inhibition causes mitochondrial dysfunction. (A) RNAi has been used to inhibit the expression of *Dnm1l* and *Mfn1* at the same time in NMVMs. Leading edge analysis based on GSEA of RNA-seq on *Dnm1l* and *Mfn1* inhibited CMs, indicating the enrichments of early heart development genes and negative enrichments of mtDNA transcription. **(B)** Scatter plots show the similar GO term enrichment profile to RNA-seq of *Dnm1l* and *Mfn1* inhibited CMs and Yap1^{GOF} hearts, indicating the accumulation of ROS and mitochondrial function.

Figure S12

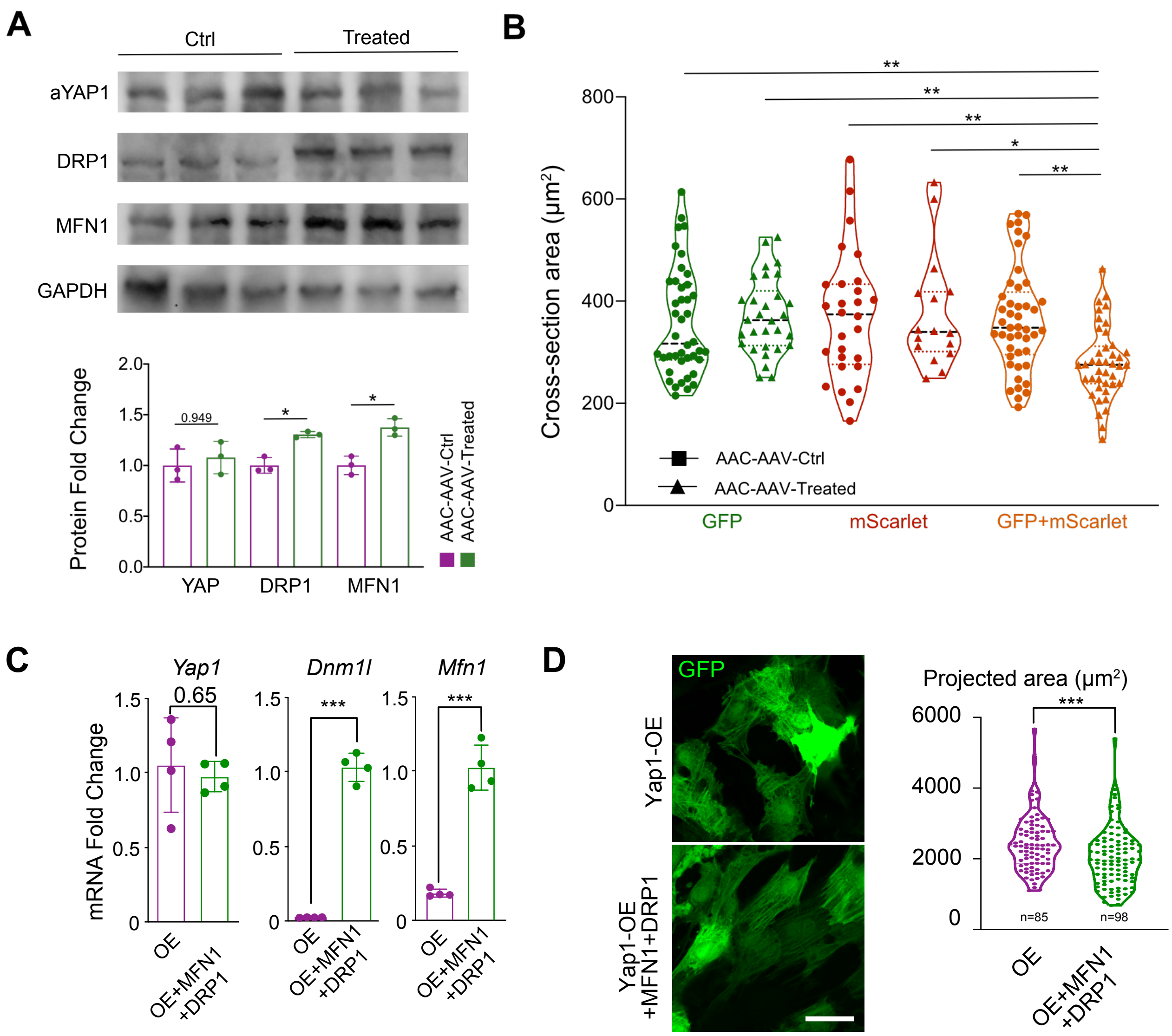

Figure 12. *Dnm1l* and *Mfn1* addback rescue hypertrophy of cardiomyocytes induced by YAP1 activation. (A) Immunoblotting for YAP, FRP1 and MFN1 after AAV derived DRP1 and MFN1 addback treatment in vivo. **(B)** Violin plots showed the reduced cardiomyocyte cross-section area in co-infected cardiomyocytes on AAC myocardial sections. **(C)** Relative expression of *Yap1*, *Dnm1l* and *Mfn1* in YAP1-overexpressing NMVMs upon MFN1 and DRP1. N = 4 biological replicates in each group. **(D)** GFP imaging and projected cell area in NMVMs upon MFN1 and DRP1 addback. GFP was a surrogate marker for NMVMs that were transduced with AdV that expressed MFN1 and DRP1. N = 3 biological replicates in each group. Scale bar, 50 μm. In statistical analysis, student's t-test was applied. *P < 0.05. **P < 0.01. ***P < 0.001. Non-significant P values in parenthesis.

Figure S13

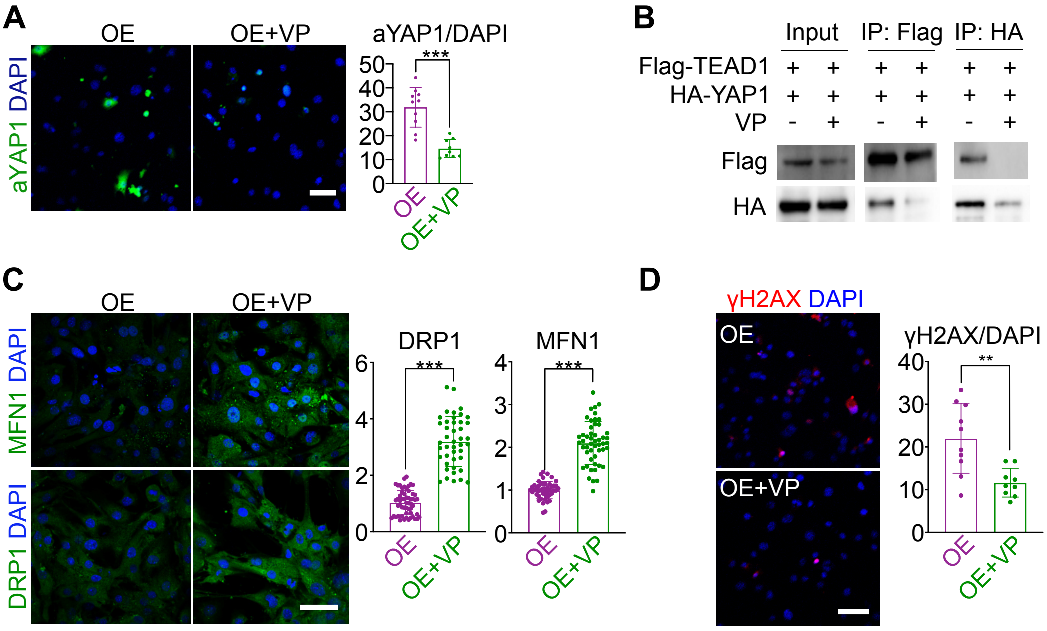

Figure S13. Verteporfin attenuates YAP1-induced DNA damages in NMVMs. (A) Immunofluorescence analysis of aYAP1 in NMVMs. **(B)** Co-immunoprecipitation of Flag-TEAD1 and HA-YAP1 in 293T cells at the presence and absence of Verteporfin. **C.** Immunofluorescence analysis of DRP1 and MFN1 in NMVMs. **D.** Immunofluorescence analysis of γH2AX in NMVMs. Scale bar, 50 μm. Mann-Whitney U-test applied, $^{**}P < 0.01$, $^{***}P < 0.001$. VP, Verteporfin

Figure S14

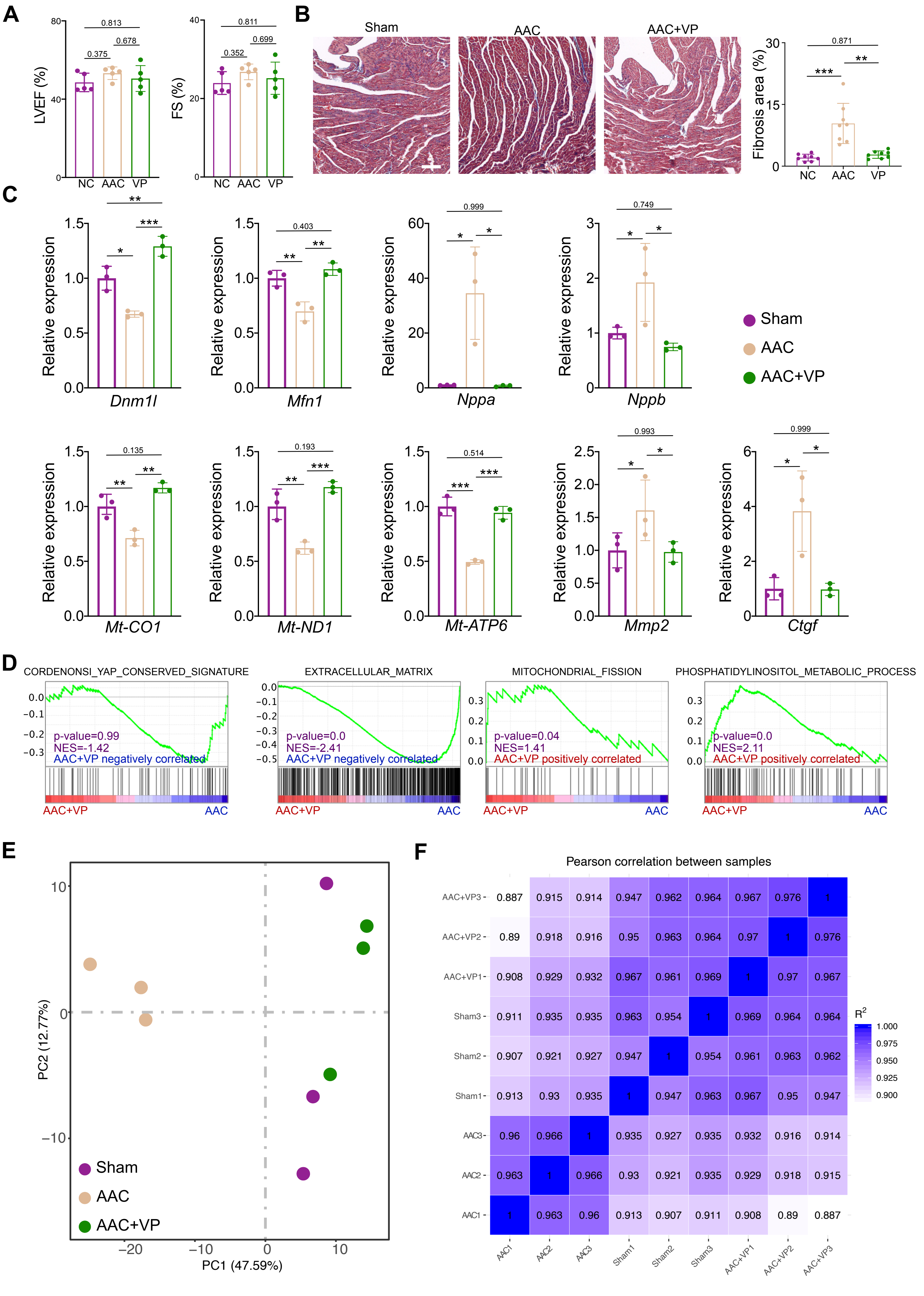
A
LVEF (%)
FS (%)
NC AAC VP
0.813
0.678
0.375
0.811
0.699
0.352
B
Sham
AAC
AAC+VP
Fibrosis area (%)
0.871
C
Relative expression
Dnm1l
Mfn1
Nppa
Nppb
Mt-CO1
Mt-ND1
Mt-ATP6
Mmp2
Ctgf
0.403
0.999
0.749
0.135
0.193
0.514
0.993
0.999
Sham
AAC
AAC+VP
D
CORDENONSI_YAP_CONSERVED_SIGNATURE
p-value=0.99
NES=-1.42
AAC+VP negatively correlated
EXTRACELLULAR_MATRIX
p-value=0.0
NES=-2.41
AAC+VP negatively correlated
MITOCHONDRIAL_FISSION
p-value=0.04
NES=1.41
AAC+VP positively correlated
PHOSPHATIDYLINOSITOL_METABOLIC_PROCESS
p-value=0.0
NES=2.11
AAC+VP positively correlated
AAC+VP
AAC
E
PC2 (12.77%)
PC1 (47.59%)
Sham
AAC
AAC+VP
F
Pearson correlation between samples
AAC+VP3 0.887 0.915 0.914 0.947 0.962 0.964 0.967 0.976 1
AAC+VP2 0.89 0.918 0.916 0.95 0.963 0.964 0.97 1 0.976
AAC+VP1 0.908 0.929 0.932 0.967 0.961 0.969 1 0.97 0.967
Sham3 0.911 0.935 0.935 0.963 0.954 1 0.969 0.964 0.964
Sham2 0.907 0.921 0.927 0.947 1 0.954 0.961 0.963 0.962
Sham1 0.913 0.93 0.935 1 0.947 0.963 0.967 0.95 0.947
AAC3 0.96 0.966 1 0.935 0.927 0.935 0.932 0.916 0.914
AAC2 0.963 1 0.966 0.93 0.921 0.935 0.929 0.918 0.915
AAC1 1 0.963 0.96 0.913 0.907 0.911 0.908 0.89 0.887
AAC1 AAC2 AAC3 Sham1 Sham2 Sham3 AAC+VP1 AAC+VP2 AAC+VP3
R2
1.000
0.975
0.950
0.925
0.900

Figure S14. Verteporfin restore AAC-induced damages in vivo. (A) The echocardiographic analysis of LVEF and FS in AAC, Verteprofin treated and sham mice. Mean ± SD. N = 6. **(B)** Masson staining on cardiac sections at the one month after Verteprofin administration and quantification of the relative fibrotic area. N = 4 hearts. Scale bar, 200 μm. **(C)** RT-qPCR analysis of cardiac remodeling and fibrotic genes in AAC hearts subjected to Verteprofin. N = 3 hearts. **(D)** GSEA analysis of RNA-seq data for hearts between AAC and AAV+VP. **(E)** PCA analysis among AAC, AAC+VP and Sham groups. **(F)** Sample correlation among AAC, AAC+VP and Sham groups. In statistical analysis, student's t-test was applied. *$P < 0.05$. **$P < 0.01$. ***$P < 0.001$. VP, Verteporfin.

Figure S15

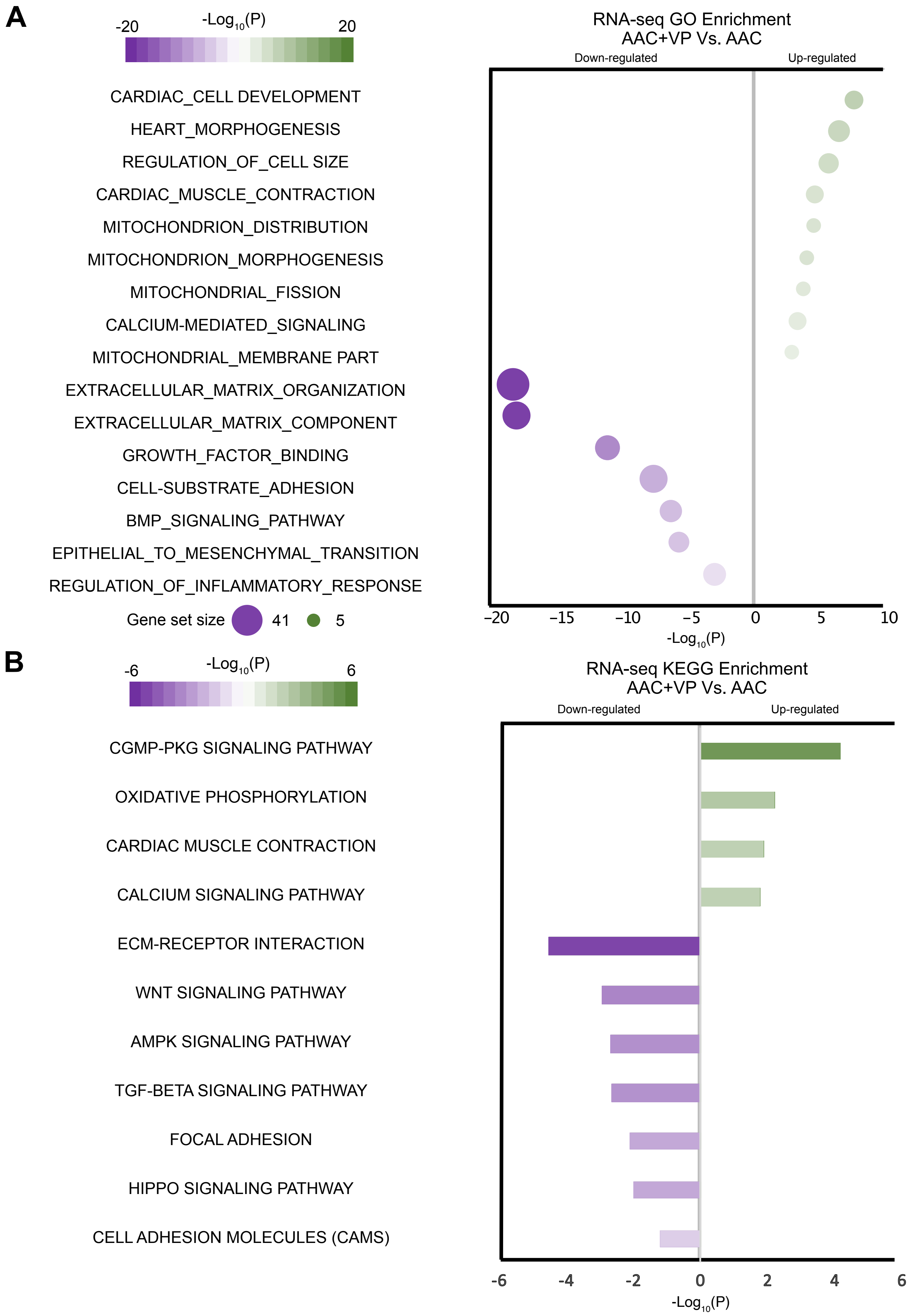

Figure S15. Verteporfin attenuates AAC induced mitochondrial impairment. (A) Scatter plots show the GO term enrichment profile to RNA-seq of AAC and AAC+VP hearts, indicating the rescue of mitochondrial and myocardial function. **(B)** Scatter plots show the KEGG term enrichment profile to RNA-seq of AAC and AAC+VP hearts, indicating the rescue of mitochondrial function and inhibition of fibrosis. VP, Verteporfin.
